# Graph-Theoretical Signature from Neural and Vascular Signals Reveals Spinal Cord Stimulation Frequency-Specific Brain Network in Disorders of Consciousness Patients

**DOI:** 10.34133/cbsystems.0539

**Published:** 2026-04-23

**Authors:** Nan Wang, Xiaoke Chai, Yifang He, Jiuxiang Song, Tianqing Cao, Qiheng He, Sipeng Zhu, Yitong Jia, Juanning Si, Yi Yang, Jizong Zhao

**Affiliations:** ^1^ Department of Neurosurgery, Beijing Tiantan Hospital, Capital Medical University, Beijing 100070, China.; ^2^ Department of Neurosurgery, Peking Union Medical College Hospital, Chinese Academy of Medical Sciences and Peking Union Medical College, Beijing 100730, China.; ^3^ China National Clinical Research Center for Neurological Diseases, Beijing 100070, China.; ^4^ School of Instrumentation Science and Opto-Electronics Engineering, Beijing Information Science and Technology University, Beijing 102206, China.; ^5^ School of Advanced Manufacturing, Nanchang University, Nanchang, Jiangxi 330031, China.; ^6^ Department of Neurosurgery, Aviation General Hospital, Beijing 100012, China.; ^7^ Brain Computer Interface Transitional Research Center, Beijing Tiantan Hospital, Capital Medical University, Beijing 100070, China.

## Abstract

**Introdution:** Spinal cord stimulation (SCS) has emerged as a promising neuromodulatory intervention for patients with disorders of consciousness (DoC). However, the identification of optimal stimulation frequencies remains a subject of ongoing debate. Although previous electroencephalography (EEG) and functional near-infrared spectroscopy (fNIRS) studies have suggested the therapeutic efficacy of 5- and 70-Hz, respectively, the integrative neurovascular mechanisms and frequency-specific network dynamics underlying these effects remain to be elucidated. **Objective and Impact Statement:** This study aims to characterize frequency-dependent network reconfiguration in DoC using simultaneous EEG-fNIRS recordings and graph theoretical analysis. By delineating distinct neurophysiological and hemodynamic signatures, our findings establish a mechanistic framework for the optimization of SCS parameters, thereby advancing personalized neuromodulation strategies for the promotion of consciousness recovery. **Methods:** This prospective trial used simultaneous EEG–fNIRS and graph theory in 16 patients with DoC undergoing multifrequency SCS at 5, 20, 70, and 100 Hz to decode frequency-specific network dynamics. Our integrated EEG–fNIRS analysis revealed 3 principal advances. First, multimodal cortical mapping via a unified anatomical atlas quantified frequency-dependent network reconfiguration, generating graph-theoretical metrics (global and nodal efficiency, characteristic path length, and clustering coefficients) from source-localized EEG (delta–gamma bands) and fNIRS (oxyhemoglobin and deoxygenated) data. Second, we identified frequency-dependent neurophysiological profiles. **Results:** Five-hertz stimulation produced acute enhancement of theta-band global network efficiency coupled with elevated gamma-band nodal efficiency in the right cingulate motor area, indicating immediate frontolimbic engagement. Conversely, 70-Hz stimulation selectively evoked delayed hemodynamic responses in the visual cortices and increased occipital hemoglobin oxygenation without concomitant EEG alterations, suggesting preferential retinotopic pathway recruitment. **Conclusion:** Multimodal EEG–fNIRS analysis elucidates frequency-specific SCS mechanisms, where 5-Hz stimulation optimizes local information integration through theta and gamma modulation, while 70-Hz enhances long-range connectivity, exposing frequency-specific neural plasticity mechanisms.

## Introduction

Disorders of consciousness (DoC), including vegetative state/unresponsive wakefulness syndrome (VS/UWS) and minimally conscious state (MCS), present substantial therapeutic challenges due to limited evidence-based interventions [1,2]. Contemporary interventions include pharmacotherapies, such as amantadine and zolpidem, and neuromodulation techniques [3]. Within the latter, noninvasive modalities such as transcranial direct current stimulation and transcranial magnetic stimulation, as well as invasive approaches such as deep brain stimulation and spinal cord stimulation (SCS), have demonstrated heterogeneous but promising effects on arousal enhancement and the reestablishment of neural connectivity [4,5]. However, standardized protocols remain elusive, and large-scale randomized trials are urgently needed to validate these interventions [6].

SCS has demonstrated potential to induce sustained neurological improvement even after stimulation cessation. Studies in patients with stroke suggest that this prolonged benefit may stem from activity-dependent plasticity, wherein residual white matter tracts undergo reorganization and growth within specific neuronal populations activated by SCS-recruited afferent pathways during rehabilitation [7–9]. Critical to harnessing this therapeutic potential is the optimization of stimulation parameters. Recent work, notably from the laboratory of Prof Grégoire Courtine, has advanced the precision of neuromodulation protocols. For instance, in mapping proprioceptive projections for upper limb muscles using spinal functional magnetic resonance imaging, muscle stimulation was applied at frequencies of 55 to 75 Hz [10], a range aligned with the optimal responsiveness of muscle spindle afferents [11]. For individuals with spinal cord injury (SCI), the same team developed a targeted epidural electrical stimulation system. In one paradigm, epidural electrical stimulation delivered at 120 Hz was evaluated for its safety and efficacy in managing hypotension, a common complication of SCI [7]. Furthermore, the ARC^EX^ therapy protocol, which uses stimulation at 30 Hz with a 10-kHz carrier frequency, has proven safe and effective in substantially improving hand and arm function—including pinch force, grip strength, and sensorimotor capacity—in patients with cervical SCI [12]. These studies underscore that specific stimulation frequencies are pivotal for engaging distinct physiological pathways and achieving targeted clinical outcomes. However, in the context of treating DoC, a similarly parameter-optimized framework for SCS remains notably underdeveloped. Here, the therapeutic target shifts from modulating spinal locomotor or autonomic circuits to influencing ascending arousal pathways and distributed forebrain networks—an application whose optimal stimulation parameters are still largely unexplored.

Clinical studies report improved Coma Recovery Scale-Revised (CRS-R) scores, tracheal decannulation rates, and electrophysiological markers in patients receiving cervical SCS [13–15]. SCS has emerged as a promising intervention for DoC, modulating thalamocortical networks and enhancing functional connectivity (FC) [16]. Functional near-infrared spectroscopy (fNIRS) studies highlighted 70 Hz for augmenting prefrontal hemodynamics and FC [17]. Electroencephalogram (EEG) studies identified 5 and 70 Hz as efficacious for enhancing cortical complexity and beta-band clustering coefficients [18,19]. Nevertheless, a consensus on the optimal stimulation frequency has yet to be reached. Parameters range widely (5 to 100 Hz) across studies, with 70 Hz frequently associated with prefrontal hemodynamic responses and beta-band EEG synchronization [16]. This variability underscores an unmet need for frequency-specific mechanistic insights to maximize therapeutic outcomes. In addition, no study has concurrently validated these effects using combined EEG–fNIRS, leaving a gap in understanding how electrophysiological and hemodynamic responses coevolve during SCS [20,21].

**Fig. 1.**
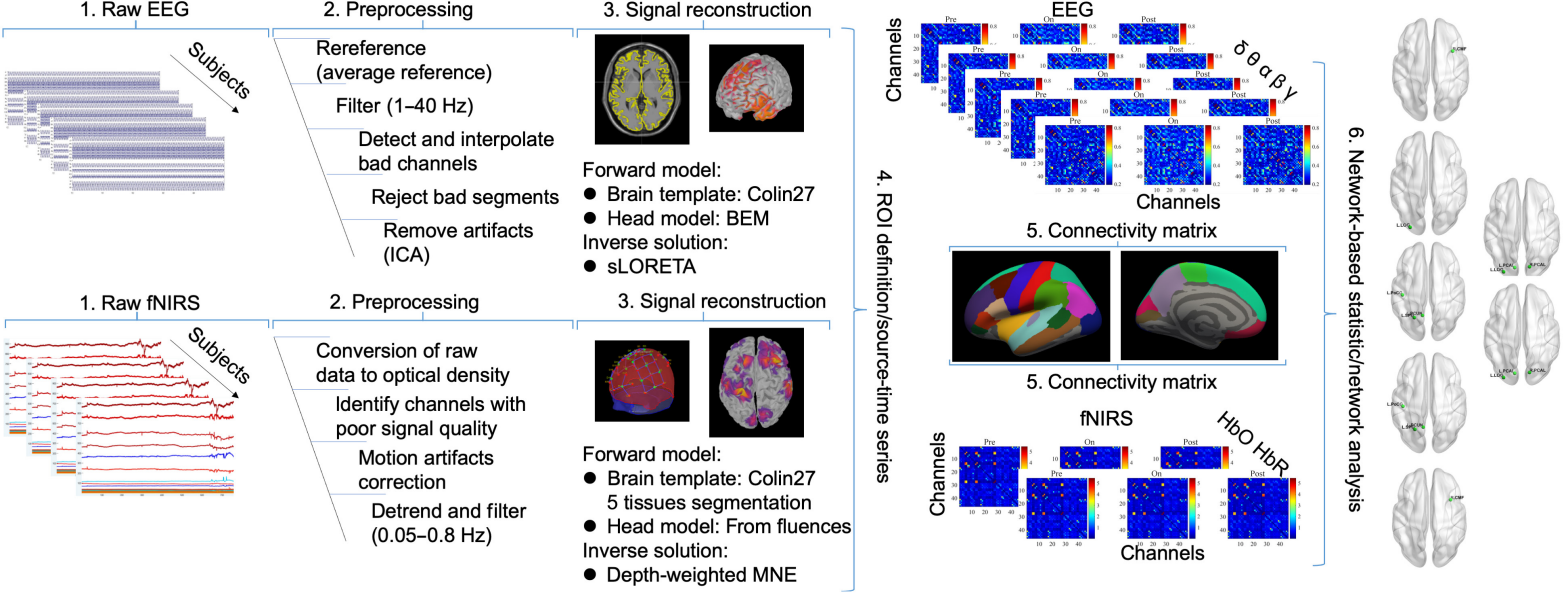
Flowchart. (1) The EEG and fNIRS data recordings for all the subjects. (2) The preprocessing steps for cleaning data. (3) The methods for the reconstruction of the signals in the source space. A BEM head model incorporating multiple tissue compartments (scalp, skull, cerebrospinal fluid, and brain) was constructed to simulate the electrical conductivity profiles across heterogeneous head tissues for EEG source localization. Cortical source current density was estimated dipole-wise using sLORETA. Forward modeling integrated Monte Carlo light transport (10^8^ photons) in a 5-layer Colin27 head model with adjoint-derived sensitivity computation, followed by Voronoi-based cortical surface projection for sulcal/gyral-resolved sensitivity mapping. wMNE with spatially adaptive regularization counteracts superficial bias for balanced cortical/deep brain source reconstruction in fNIRS. (4) The EEG and fNIRS source-time series were mapped in the same 3-dimensional space using an atlas-based approach (Desikan–Killiany). (5) FC (Pearson’s correlation) estimates the statistical coupling between each ROI of the reconstructed time series. (6) The topology of brain networks captured by the 2 techniques was compared through graph theoretical approaches.

Therefore, the current study is motivated by the critical need to bridge this translational gap. We introduce a simultaneous EEG–fNIRS framework during SCS, leveraging graph-theoretical metrics (e.g., clustering coefficient and nodal efficiency) to quantify network integration across electrophysiological and hemodynamic domains. This approach integrates multimodal data to decode frequency-specific network reconfiguration. Moreover, this work investigates the coupled dynamics between cortical oscillatory activity and prefrontal hemodynamic responses elicited by SCS. Importantly, it leverages multimodal neurophysiological convergence metrics to quantify therapeutic efficacy beyond conventional behavioral scales (Fig. 1). By integrating these insights, we define a frequency-optimized SCS paradigm for DoC, paving the way for personalized neuromodulation strategies.

## Materials and Methods

### Patients with DoC

This prospective study included 16 patients diagnosed with DoC who received SCS as clinical treatment. Based on the CRS-R scores, they were diagnosed as being in the MCS group (4 subjects) or the VS/UWS group (12 subjects). These patients exhibited prolonged DoC for at least 28 d post-brain injury, with no severe brain structural damage or abnormalities. Preoperative consciousness status scores were CRS-R T_0_. Follow-up assessments were conducted at 1 month. The CRS-R was used to assess consciousness levels post-SCS surgery. To ensure reliability and minimize subjectivity, all CRS-R evaluations in our study were conducted independently by 2 attending physicians, each with over 5 years of specialized experience in managing DoC. For patients whose condition deteriorated because of complications, we considered the data from the best available follow-up assessment. Table 1 provides detailed patient information. Patients primarily received rehabilitation therapy according to clinical guidelines. Other interventions were tailored to the patient’s specific condition, determined on the basis of the physician’s assessment results, and followed the recommended protocol. These interventions typically included routine rehabilitation therapy (such as active and passive limb movements) and supportive therapy (including skin management, complication control, and nutritional support).

**Table 1.** Demographic data and clinical characteristics of patients

Variable	Value
Age, years, mean (SD)	49.38 (14.00)
Sex/%	
Female	3 (18.8)
Male	13 (81.3)
Etiology/%	
Anoxia	3
Trauma	6
Stroke	7
Diagnosis/%	
MCS	4
VS/UWS	12
Interval since injury, months, mean (SD)	10.25 (10.08)
CRS-R scores, mean (SD)	9.65 (4.60)

Abbreviations: VS/UWS, vegetative state/unresponsive wakefulness syndrome; MCS, minimally conscious state; CRS-R, Coma Recovery Scale-Revised.

The clinical dataset analyzed in this study was obtained from patients with DoC who received clinical treatment at the Department of Neurosurgery, Beijing Tiantan Hospital. This study was approved by the Ethics Committee of Beijing Tiantan Hospital, Capital Medical University (ethics review number: KY2024-043-03) and the Chinese Clinical Trial Registry (ChiCTR2400085830). The patients’ parents or legal guardians signed informed consent forms after receiving detailed information and discussing the treatment process, ensuring that the patients’ parents or legal guardians were fully informed and consented to the treatment and potential prospective research aspects. The consent forms provided comprehensive details about the treatment process, outlining the surgical procedures, associated risks, and the possibility of treatment being completely ineffective. All clinical data collected in the study were used for clinical treatment. No additional examinations were conducted for research purposes.

### Blinding

The collection and analysis of patient data were conducted by different research teams using a completely blinded method. Researchers from the neurosurgery department collected clinical data but were unaware of the brain connectivity characteristics of patients with DoC or the specific stimulation parameters. Researchers analyzed spinal cord electrical stimulation discharge signals, light signals representing metabolic activity, and brain connectivity. Before completing all discharge signal features, light signal, and brain connectivity analyses, they did not know the patients’ conscious state or SCS outcomes.

### Experimental paradigm

**Fig. 2.**
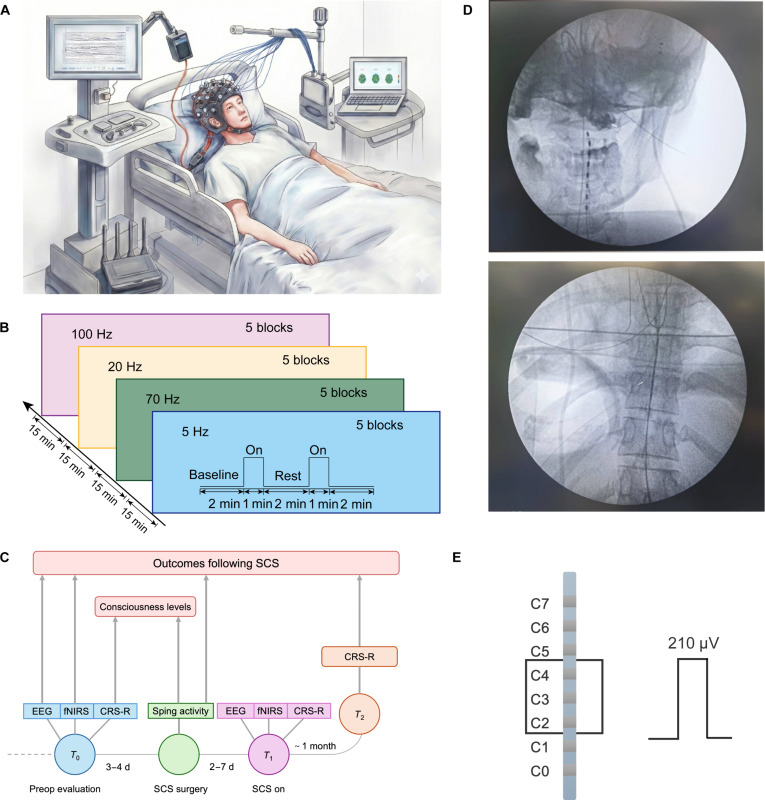
Experimental paradigm diagram. (A) Configuration of the experiment. (B) Frequency-specific SCS protocol for patients with DoC. Multimodal neuroimaging sequence: (1) 15-min resting-state EEG–fNIRS baseline; (2) 5-Hz SCS trial: 2-min prestimulation → 1-min stimulation (5-Hz biphasic pulses) → 2-min poststimulation rest (5 cycles); (3) 15-min washout; (4) sequential frequency trials (70, 20, and 100 Hz) replicating 5-Hz protocol. Fifteen-minute intertrial intervals eliminated carryover effects. (C) Timeline illustrating the procedure throughout different phases of the study. CRS-R scores, EEG, and fNIRS were obtained from all patients at baseline (T0; 3 to 4 d postoperatively) and during initial stimulator activation (T1). The CRS-R scores were also assessed at the 1-month follow-up (T2) to evaluate chronic recovery outcomes. (D) Intraoperative fluoroscopic validation of epidural lead placement for SCS in DoC. Anteroposterior view demonstrating C2 to C4 level electrode positioning with bilateral equidistant spacing from the spinal midline (<1.5-mm deviation) (top). Lateral view confirming dorsal epidural placement depth and parallel alignment to spinal curvature. Targeting accuracy validated against preoperative neuronavigational planning (bottom). (E) Physiologically titrated SCS parameter configuration for consciousness restoration. Waveform schematics. Biphasic cathodic-leading pulses (pulse width, 210 μV) with amplitude titrated to sensorimotor threshold, defined as the minimal voltage eliciting visible upper limb myoclonus (2 to 6 V).

The experimental configuration is shown in Fig. 2A. To assess potential associations between brain connectivity dynamics, neural reorganization, and recovery outcomes after SCS, we analyzed perioperative neurological features, including electrophysiological discharges and metabolic profiles, as well as the CRS-R scores (Fig. 2C). For each patient with DoC, CRS-R was rigorously administered 3 to 4 d before SCS implantation. Multimodal assessment included CRS-R reassessment 2 to 7 d after electrode implantation and 1 month after SCS programmed to quantify the evolution of the state of consciousness. The specific SCS parameters, frequencies of 5, 20, 70, and 100 Hz, are summarized in Fig. 2B. Dual-modality synchronized EEG and fNIRS devices were used to collect data from patients. Following the initial week of undergoing SCS, after extensive titration tests on stimulation frequency and intensity, the optimal geometry of each electrode contact was determined on the basis of the limitation of noticeable arousal effects and visible side effects. Subsequently, all patients underwent sequential stimulation with groups A to D. Each frequency was activated for 1 min of SCS stimulation, followed by 2 min of deactivation, repeated 5 times. After each frequency cycle, a 15-min rest period is observed. The pulse width is set to 210 μV. The voltage is adjusted until the patient’s limb exhibits mild tremors [16]. Group A’s stimulation parameters are set to a frequency of 5 Hz, group B’s to 70 Hz, group C’s to 20 Hz, and group D’s to 100 Hz. Electrode localization was confirmed by postoperative x-ray coregistration (Fig. 2D) and detailed schematics of pulse-width modulation for each stimulation (Fig. 2E).

### Multimodal electrophysiological and hemodynamic acquisition

Neural signatures were acquired using a synchronized EEG–fNIRS platform during both resting-state and SCS stimulation epochs to integrate EEG–fNIRS interrogation of SCS-induced neural dynamics in DoC. Photoelectric temporal locking enabled precise quantification of neurovascular coupling (NVC) dynamics, establishing multimodal biomarkers for SCS parameter optimization in consciousness restoration.

EEG signals were acquired using a wireless EEG recording system (NeuroHUB, Neuracle, Changzhou, China). Data were recorded at a sampling rate of 1,000 Hz via the manufacturer’s proprietary software (Neuracle Collect). Electrode placement for EEG signal acquisition adhered to the international standard 10-10 system, utilizing a montage of 32 channels [22].

The fNIRS data were acquired using the NirSmartII-3000A system (Jiangsu Danyang Huichuang Medical Equipment Co. Ltd.). Two wavelengths, 730 and 850 nm, were used to detect changes in oxyhemoglobin (HbO), deoxygenated hemoglobin (HbR), and total hemoglobin concentrations in the brain in real time. The fNIRS system consisted of 22 sources and 15 detectors, totally generating 45 optical channels, with a source–detector distance of 3.0 cm. The “S” and “D” circles represent the light source and detector, respectively, and the connecting lines with numbers indicate the optical channels of fNIRS (Fig. S1). The fNIRS system sampling frequency was 11 Hz.

### EGG and fNIRS acquisition and preprocessing

Raw EEG data underwent the following preprocessing pipeline in EEGLAB v2025.0.0 [23]: (a) Data conversion and channel localization. Raw data were converted to EEGLAB-compatible. Format was set. EEG channels were extracted and coregistered to standard Montreal Neurological Institute space using the international 10-10 electrode placement system for spatial normalization. (b) Downsampling. Data were downsampled to 256 Hz to reduce computational load while preserving signal integrity. (c) Bandpass filtering. A bandpass filter (1 to 45 Hz) was applied to attenuate low-frequency drifts and high-frequency noise (e.g., muscle artifacts and line interference), retaining physiologically relevant EEG bands. (d) Epoching and artifact rejection. Continuous data were segmented into 2-s epochs. Epochs contaminated by prominent artifacts (e.g., motion and electromyographic interference) were manually identified and removed. Channels with persistent poor signal quality (e.g., flatlines and excessive noise uncorrected by filtering) were identified via visual inspection and statistical outlier detection (signal variance and kurtosis). These were interpolated using spherical spline interpolation. The number of interpolated channels per recording is detailed in the column “nBadCh” in Table S1. Across all datasets, an average of 1.08 ± 1.77 (means ± SD) channels were interpolated, representing approximately 3.4% of the total 32 channels per recording. (e) Referencing was referenced to the common average reference to mitigate reference electrode bias. (f) Independent component analysis (ICA) denoising. (g) ICA decomposed data into maximally independent components. Components representing biological (e.g., ocular movements, blinks, and muscle activity) or nonbiological artifacts (e.g., line noise and equipment artifacts) were identified on the basis of spatial topography, time-course characteristics, and power spectral profiles and subsequently removed. (h) Post-ICA artifact rejection. A final amplitude thresholding step (−100 to +100 μV) was applied to exclude residual artifact-contaminated epochs, ensuring robust signal quality for downstream analysis. (i) Data retention. The final clean data duration after all rejection steps is listed as “RetainDuration” in Table S1. The overall mean data retention rate was 85.7% of the original recording length (“TotalDuration”).

Preprocessing was performed using the Brainstorm [24] toolbox with the NIRSTORM [25] extension, implementing the following pipeline. First, raw light-intensity signals were converted to optical density units to better reflect hemodynamic changes associated with HbO and HbR concentrations in cerebral tissue. Second, temporal derivative distribution repair [26], a statistical approach leveraging temporal derivative distributions, was applied to detect and correct abrupt signal shifts induced by head motion, minimizing contamination from movement artifacts. Third, a third-order zero-phase Butterworth infinite impulse response bandpass filter (0.01 to 0.1 Hz) was implemented to eliminate noise: High-pass filtering (0.01 Hz) removed slow instrumental drifts, while low-pass filtering (0.1-Hz cutoff) attenuated physiological high-frequency noise including Mayer waves (~0.1 Hz), cardiac oscillations (1.6 to 1.8 Hz), and respiratory fluctuations (0.2 to 0.3 Hz). All preprocessing steps ensured physiological relevance while minimizing contamination from extraneous sources.

Processed EEG epochs and fNIRS time-series data were precisely aligned using shared stimulus onset markers. For correlational and network analyses requiring uniform temporal resolution, the fNIRS data were temporally interpolated to match the EEG epoch timestamps. This synchronized multimodal dataset was used for all subsequent analyses of the brain network.

### Source localization

EEG source localization was performed using the Brainstorm toolbox [24]. First, a head volume conduction model was constructed for EEG via OpenMEEG [10], implementing the boundary element method (BEM) [27] on the Colin27 template brain. This model incorporated geometric and electrical conductivity properties of scalp, skull, and brain tissue layers to simulate current propagation. Cortical sources were modeled as 15,000 dipoles distributed across the cortical surface mesh. From this, a lead field matrix was generated to map theoretical scalp potentials from each dipole to EEG electrodes, solving the forward problem. Second, the standardized low-resolution brain electromagnetic tomography (sLORETA) algorithm [28] was applied to the forward solution to estimate current source density. As a linear minimum-norm inverse solution, sLORETA enhances spatial resolution through source-space standardization. It assumes a zero-mean, unit-covariance prior distribution to achieve noise-robust 3-dimensional reconstruction of neural activity while constraining source localization stability.

fNIRS source localization was performed using Brainstorm [24] and NIRSTORM [25] toolbox. First, a forward model was constructed via Monte Carlo simulation (10^8^ photons) on the Colin27 template brain’s 5-tissue segmentation (scalp, skull, cerebrospinal fluid, and gray/white matter) to compute photon flux distributions between sources and detectors. Sensitivity matrices were derived using the adjoint method under the Rytov approximation, multiplying source/detector photon flux rates to quantify channel-wise sensitivity at each voxel. These volumetric sensitivity values were projected to a 15,000-vertex cortical mesh using Voronoi tessellation interpolation, preserving sulcal/gyral anatomy for high-fidelity surface mapping. Second, depth-weighted minimum norm estimation (wMNE) [7–9] solved the inverse problem. This approach incorporated depth-weighting factors to counteract superficial source bias inherent in traditional MNE, enabling spatially uniform reconstruction of neural activity, particularly enhancing accuracy in clinically relevant deep brain regions.

To enable cross-modal comparisons, EEG and fNIRS source time-series were mapped to a unified anatomical space using the Desikan–Killiany cortical atlas [29]. First, given the fNIRS’s limited spatial coverage, we adapted the atlas to retain only regions of interest (ROIs) within optode sampling areas (46 ROIs preserved from the original 68-region parcellation). This ensured spatial correspondence between modalities. Subsequently, for each ROI, EEG signals were bandpass-filtered into canonical oscillatory bands: delta (1 to 4 Hz), theta (4 to 8 Hz), alpha (8 to 15 Hz), beta (15 to 25 Hz), and gamma (25 to 45 Hz). fNIRS sources were converted to hemodynamic responses via the modified Beer–Lambert law [30], yielding ΔHbO and ΔHbR concentration changes to quantify regional cerebral blood flow and metabolic activity (Table 2).

**Table 2.** EEG and fNIRS common 46 ROIs

Number	Brain region	Abbreviations for brain regions	Brain lobe	Abbreviation for brain lobe
1	Bankssts L	L.BSTS	Temporal	T
2	Bankssts R	R. BSTS	Temporal	T
3	Caudal middle frontal L	L.CMF	Frontal	F
4	Caudal middle frontal R	R.CMF	Frontal	F
5	Cuneus L	L.CUN	Occipital	O
6	Cuneus R	R.CUN	Occipital	O
7	Frontal pole L	L.FP	Prefrontal	PF
8	Frontal pole R	R. FP	Prefrontal	PF
9	Inferior parietal L	L.IPL	Parietal	P
10	Inferior parietal R	R.IPL	Parietal	P
11	Insula L	L.INS	Temporal	T
12	Insula R	R.INS	Temporal	T
13	Lateral occipital L	L.LOG	Occipital	O
14	Lateral occipital R	R.LOG	Occipital	O
15	Medial orbitofrontal L	L.MOF	Prefrontal	PF
16	Medial orbitofrontal R	R.MOF	Prefrontal	PF
17	Middle temporal L	L.MTG	Temporal	T
18	Middle temporal R	R.MTG	Temporal	T
19	Paracentral L	L.PCL	Central	C
20	Paracentral R	R.PCL	Central	C
21	Pars opercularis L	L.POG	Frontal	F
22	Pars opercularis R	R.POG	Frontal	F
23	Pars orbitalis L	L.PO	Prefrontal	PF
24	Pars orbitalis R	R.PO	Prefrontal	PF
25	Pars triangularis L	L.PTRI	Frontal	F
26	Pars triangularis R	R. PTRI	Frontal	F
27	Pericalcarine L	L.PCAL	Occipital	O
28	Pericalcarine R	R.PCAL	Occipital	O
29	Postcentral L	L.PoCG	Central	C
30	Postcentral R	R.PoCG	Central	C
31	Precentral L	L.PreCG	Central	C
32	Precentral R	R.PreCG	Central	C
33	Precuneus L	L.PCUN	Parietal	P
34	Precuneus R	R.PCUN	Parietal	P
35	Rostral middle frontal L	L.RMF	Frontal	F
36	Rostral middle frontal R	R.RMF	Frontal	F
37	Superior frontal L	L.SFG	Frontal	F
38	Superior frontal R	R.SFG	Frontal	F
39	Superior parietal L	L.SPG	Parietal	P
40	Superior parietal R	R.SPG	Parietal	P
41	Superior temporal L	L.STG	Temporal	T
42	Superior temporal R	R.STG	Temporal	T
43	Supramarginal L	L.SMG	Parietal	P
44	Supramarginal R	R.SMG	Parietal	P
45	Transversetemporal L	L.TRT	Temporal	T
46	Transversetemporal R	R.TRT	Temporal	T

L, left; R, right.

### FC analysis

FC was assessed using the phase locking value (PLV) across 5 canonical frequency bands (delta to gamma). Following bandpass filtering and Hilbert transformation of source-space signals, we calculated PLV to quantify interregional phase synchronization independent of amplitude [31,32]. We specifically selected PLV for 2 key advantages in DoC pathophysiology: First, its insensitivity to amplitude fluctuations mitigates volume conduction confounds common in pathological states [33]. Second, unlike coherence-based metrics, PLV remains robust without assuming stationarity, making it ideal for capturing the transient, nonlinear dynamics of resting-state EEG in patients with severe brain injury. Finally, band-specific PLV matrices were constructed to characterize SCS-induced network reconfiguration [34].

FC analysis of fNIRS was quantified using hemodynamic correlation metrics. Pearson correlation coefficients were computed between all channel pairs for ΔHbO and ΔHbR time series within the 0.01- to 0.1-Hz band, measuring interregional synchronization. This generated correlation matrices where each element represented FC strength (*r* value) between 2 brain regions. What’s more, Fisher’s *r*-to-*Z* transformation normalized correlation distributions to enhance parametric statistical sensitivity. The resultant *Z*-score matrices reflected final FC strength, with higher *Z*-values indicating stronger low-frequency oscillatory coupling.

### Brain network analysis based on graph theory

Functional brain networks were characterized using the Brain Connectivity Toolbox [35]. Binary adjacency matrices were generated via proportional thresholding across a sparsity range of 0.09 to 0.5 (step size, 0.01), with the lower bound (0.09) determined to minimize fragmented components while preserving network topology [36]. For each sparsity level, we computed a comprehensive set of global and nodal graph-theoretical metrics, including the small-world index (SWI; *σ*), clustering coefficient (CC), characteristic path length (CPL), global and local efficiency (Eglobal), as well as centrality measures (degree and betweenness centrality). The small-world properties of the real networks were validated by comparison with 100 randomized null networks generated for each sparsity level. To obtain a connection-density-independent characterization of network topology, the final value for each metric was calculated as the area under the curve across the entire sparsity range. The detailed mathematical definitions of all computed metrics are provided in the Supplementary Materials. Statistical analyses were then performed on these integrated metric values to assess the effects of SCS on the global and regional architecture of functional brain networks in patients with DoC. The detailed methods are available in the **Supplemental Method**.

### Statistical analysis

#### Statistical comparison of FC

The differences of FC between groups were assessed using the network-based statistic [37] method, a topological approach that controls family-wise error rate while identifying statistically significant interconnected subnetworks. First, paired *t* tests (*P* < 0.001) screened all edges across connectivity matrices, retaining candidate differential edges. Second, graph-theoretical connectivity principles clustered these edges into spatially contiguous components (subnetworks). Third, component size was quantified as its aggregate edge count. Fourth, permutation testing (10,000 iterations) determined significance. Maximum component size null distributions were generated under random group assignments. A component survived correction if its size exceeded the 95th percentile of this null distribution (component-level *P* < 0.05/3). The Bonferroni-adjusted threshold (*P* < 0.05/3) accounted for 3 independent network-based statistic analyses (ΔHbO, ΔHbR, and EEG PLV matrices) [38].

#### Statistical comparison of graph theory

This study used one-way repeated-measures analysis of variance for statistical analysis of the relevant data. To control the risk of false positives resulting from multiple comparisons, in the main effects analysis, the false discovery rate (FDR) correction method was used for multiple comparison correction in the within-group comparisons of the 46 brain region node attributes. In the post hoc tests, the Bonferroni correction method was used to further control the significance level of pairwise comparisons between groups.

## Results

### Frequency-specific modulation of global network topology

To investigate frequency-dependent neuromodulation of whole-brain FC, we computed graph-theoretical global metrics, including SWI, CC, CPL, and Eglobal, across canonical frequency bands. Significant network reconfiguration was selectively observed at 5- and 70-Hz stimulation, while 20- and 100-Hz stimulation elicited no detectable changes across metrics.

**Fig. 3.**
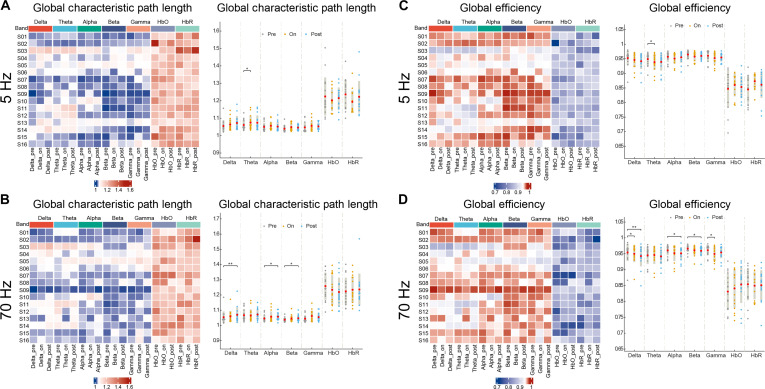
Frequency-specific modulation of global network topology during SCS. (A) CPL dynamics across EEG frequency bands (delta, theta, alpha, beta, and gamma) and fNIRS hemodynamics (ΔHbO and ΔHbR) during 5-Hz stimulation. Heatmaps show CPL values for each subject (*n* = 16) in the prestimulation (pre), on-stimulation (on), and poststimulation (post) phases. Asterisks denote significant phase-to-phase differences (paired *t* test, FDR-corrected, *P* < 0.05). (B) Eglobal changes during 5-Hz stimulation. Heatmaps show global efficiency values for each subject in the pre-, on, and postphases. Subject group-averaged (*n* = 16) highlights an acute increase in theta-band efficiency during the on-stimulation phase. Significant on- versus prestimulation differences are marked (paired *t* test, FDR-corrected, *P* < 0.05). (C) CPL reorganization during 70-Hz stimulation. Subject group-averaged reveals sustained poststimulation elevations in the delta, alpha, and gamma bands. Asterisks indicate significant changes relative to prestimulation (paired *t* test, FDR-corrected, *P* < 0.05). (D) Delayed optimization of consciousness-relevant networks following 70-Hz stimulation. Individual-level heatmaps display prestimulus versus on-stimulus versus poststimulus changes in global efficiency for EEG frequency bands (delta, theta, alpha, beta, and gamma) and fNIRS hemodynamics (ΔHbO and ΔHbR). Group-level results reveal acute gamma-band facilitation during stimulation (on versus pre) and delayed delta/alpha/beta-band optimization poststimulation (post versus pre) . All panels: Paired *t* test, FDR-corrected; *P < 0.05 and **P < 0.01.

Theta-band networks exhibited increased CPL during stimulation at 5-Hz stimulation (on versus pre, *P* < 0.01), indicating reduced integration efficiency (Fig. 3A). Conversely, theta Eglobal increased (on versus pre, *P* < 0.01), suggesting compensatory long-range synchronization (Fig. 3C). For 70-Hz stimulation, consciousness-relevant bands aligned with the arousal-brain connectivity model [39] showed distinct dynamics: Delta, alpha, and beta bands displayed prolonged CPL elevation poststimulation (post versus pre, *P* < 0.05) in Fig. 3B. The Eglobal of delta, alpha, and beta increased poststimulation (post versus pre, *P* < 0.05), indicating delayed network optimization (Fig. 3D). The gamma band showed acute efficiency enhancement during stimulation (on versus pre, *P* < 0.05), revealing immediate local circuit facilitation.

While the absence of HbO and HbR changes despite the Eglobal of EEG at 5- and 70-Hz SCS (*P* > 0.05). fNIRS revealed frequency-specific NVC. HbO decreased during stimulation (pre versus post, *P* < 0.05) without HbR changes in 100-Hz SCS, suggesting uncoupled metabolic demand (Fig. S2). Twenty- and 100-Hz stimulation elicited no significant changes in CPL and Eglobal (*P* > 0.05). This nonlinear response implies frequency-selective gating, with 5 and 70 Hz as critical thresholds for thalamocortical engagement [40]. These global dynamics originate from hub-specific reorganization. Theta inefficiency at 5 Hz likely reflects degraded connectivity in frontoparietal hubs (e.g., superior parietal gyrus), where compensatory centrality may arise. Delayed alpha and beta efficiencies at 70 Hz implicate the precuneus and superior frontal gyrus, which are the key nodes for consciousness recovery. Gamma dissociation suggests preferential modulation of the insula and the middle temporal gyrus (MTG). We next quantifydegree centrality (DC), betweenness centrality (BC), and nodal local efficiency (NElocal) across 46 ROIs to identify precision neuromodulation targets.

### Frequency-specific NVC in functional networks

**Fig. 4.**
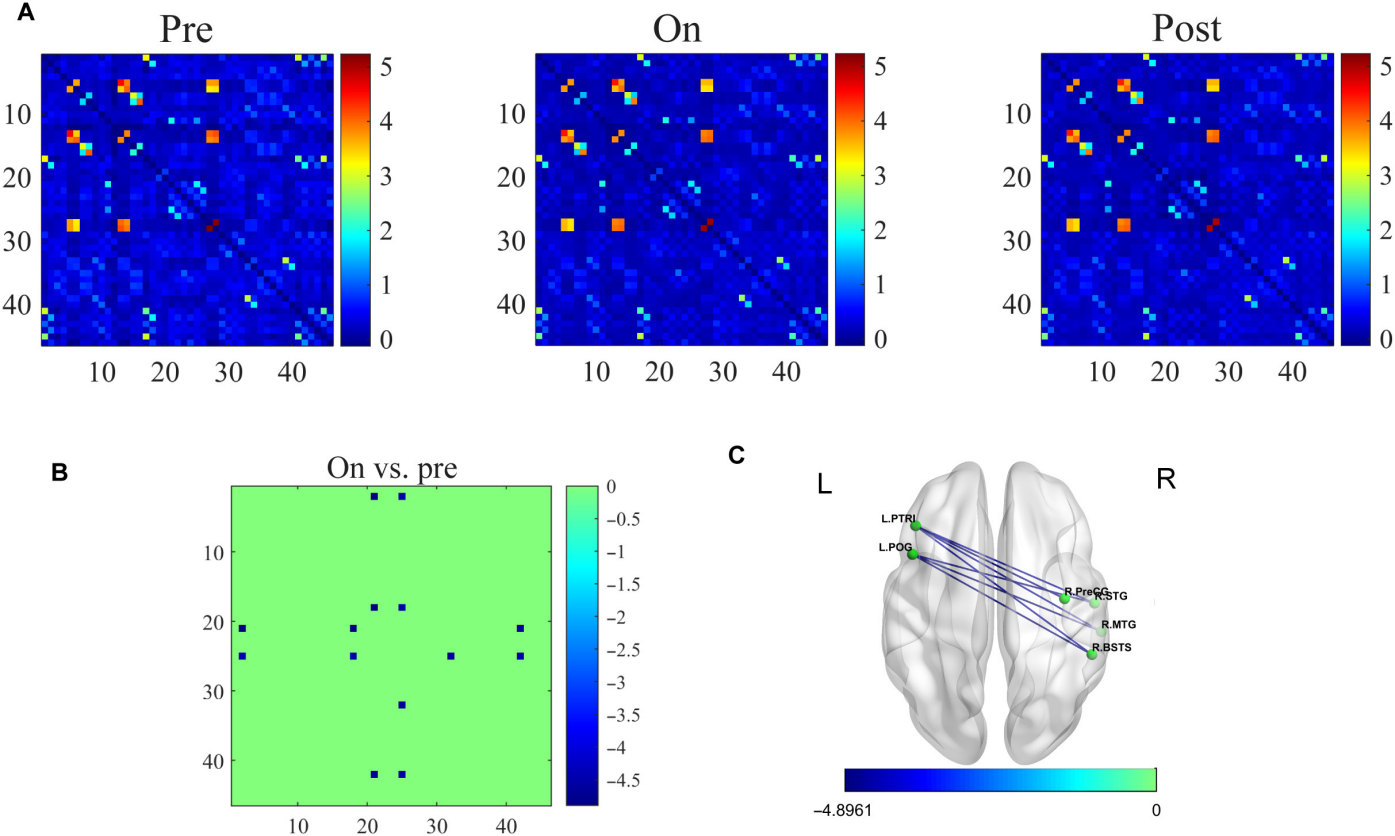
Spatial patterns of the FC in 5 Hz. (A) FC maps for pre, on, and post. (B) Significant between-condition differences in HbR-based FC. The figure displays edges showing significant changes in HbR connectivity between the pre-stimulation baseline (pre) and the on-stimulus interval (on) in patients with DoC (paired t test, FDR-corrected, *P* < 0.001). (C) Brain map of T-valued significant edges for HbR connectivity changes (pre vs on). This figure presents a cortical surface map where each edge that significantly differed between pre and on-stimulus intervals is color-coded according to its T-value. The color of each edge in the figure represents the magnitude of the T-value. A T-value less than 0 indicates lower connectivity strength in the on-stimulus condition compared with the pre-stimulation baseline, whereas a T-value greater than 0 indicates higher connectivity strength in the on-stimulus condition. All edges shown reached statistical significance (paired t-test, component-level corrected *P* < 0.001).

To investigate frequency-dependent NVC during SCS, we maintained standardized parameters (pulse width, 210 μs; interstimulus interval, 2 min) and analyzed pre-/on-/post-stimulus epochs. fNIRS FC analysis revealed a marked increase in HbR increased during 5-Hz stimulation (Fig. 4A) in 3 critical pathways. Between the right banks of the superior temporal sulcus, a hub for visual-social processing, and the left pars opercularis (POG), which mediates syntactic computation, forming a visual–language integration circuit. Connecting the right MTG, critical for lexical-semantic storage, and the left pars triangularis (PTRI), involved in semantic retrieval, facilitates auditory-semantic binding. Linking left PTRI with the right superior temporal gyrus (STG), essential for auditory-phonetic decoding, enables cross-hemispheric semantic-auditory integration. These connections constitute a frontotemporal integration subnetwork, with enhanced FC indicating SCS-potentiated communication in DoC.

**Fig. 5.**
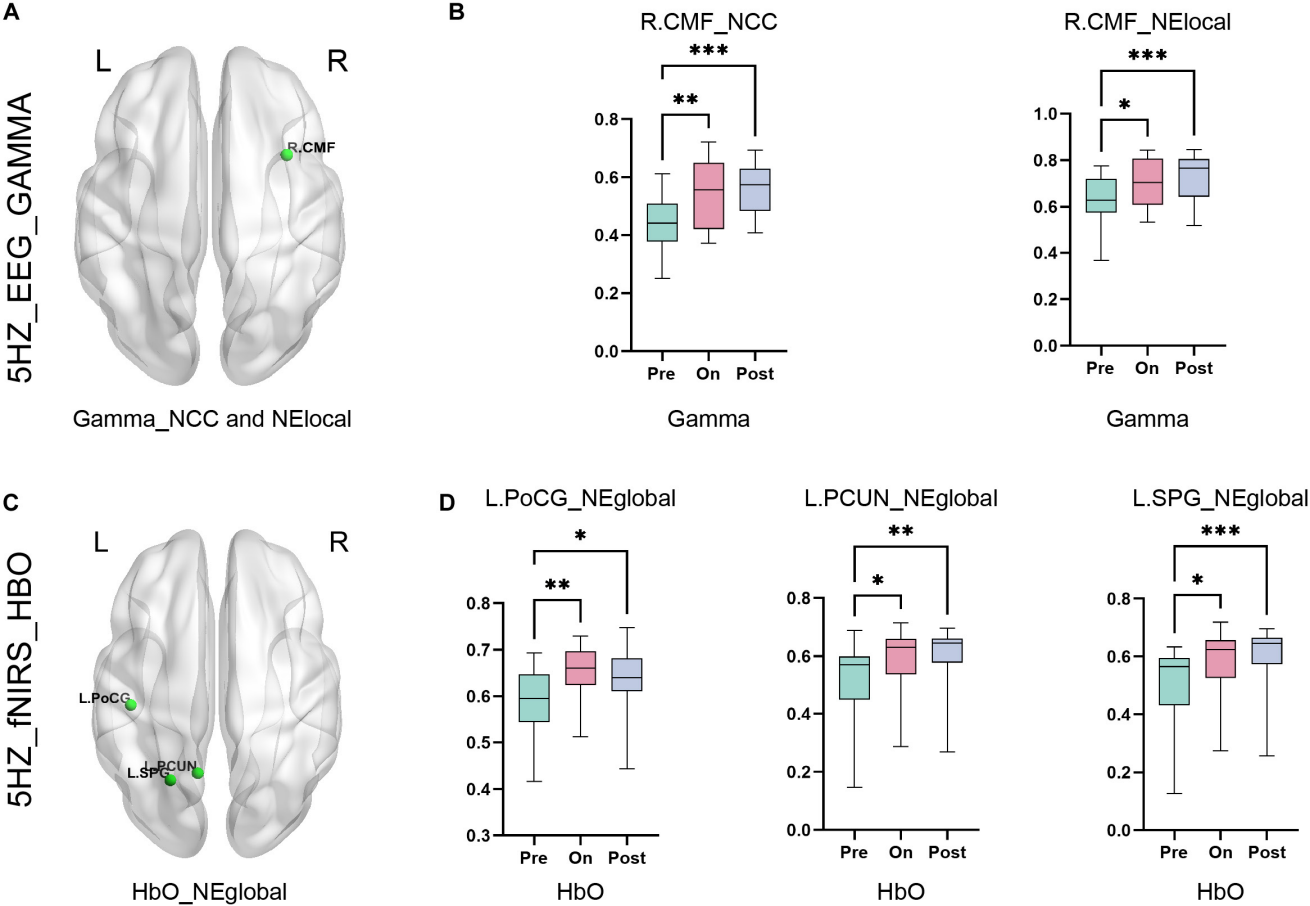
Differential neural responses to 5-Hz stimulation across electrophysiological and hemodynamic metrics. (A) Topographical maps illustrating brain regions with significant differences in NCC and NElocal within the gamma band during 5-Hz stimulation. (B) Statistical plots of NCC and NElocal changes. NCC exhibited significant differences between prestimulus versus on-stimulus (*P* < 0.01) and prestimulus versus poststimulus (*P* < 0.001) phases. NElocal showed parallel differences (pre–on, *P* < 0.05; pre–post, *P* < 0.001). Both metrics localized to the right cingulate motor area (R_CMF). (C) fNIRS-derived maps of HbO-based NElocal differences during 5-Hz stimulation. (D) Statistical plots of HbO-NElocal changes. Significant pre–on/pre–post differences were observed in the L_PoCG, L_PCUN, and L_SPG. All panels: FDR-corrected; error bars denote SEM; *P < 0.05, **P < 0.01, and ***P < 0. 001.

Comparative analysis demonstrated that selective HbR reduction in frontotemporal hubs (*P* < 0.001) without HbO changes at 5 Hz (*P* > 0.05) (Fig. 4B). 4 While no statistically significant alterations in FC were detected at 20, 70, or 100 Hz, low-frequency (5 Hz) SCS elicited substantial enhancement of prefrontal–temporal connectivity. This effect is likely mediated by frequency-dependent NVC, as evidenced by HbR dynamics (Fig. 4C). Node-level analysis at 5 Hz demonstrated frequency-specific hemodynamic alterations. HbO-derived NElocal exhibited significant increases in the L_Postcentral (L_PoCG), L_Precuneus (L_PCUN), and R_Parietal_Sup (R_SPG) during pre- and on-, pre- and poststimulation phases relative to baseline (*P* < 0.01) in Fig. 5A and B. Notably, EEG-derived gamma band metrics showed analogous changes (*P* < 0.01), indicating distinct modality-specific responses.

### NVC to specific stimulation at 70 Hz

**Fig. 6.**
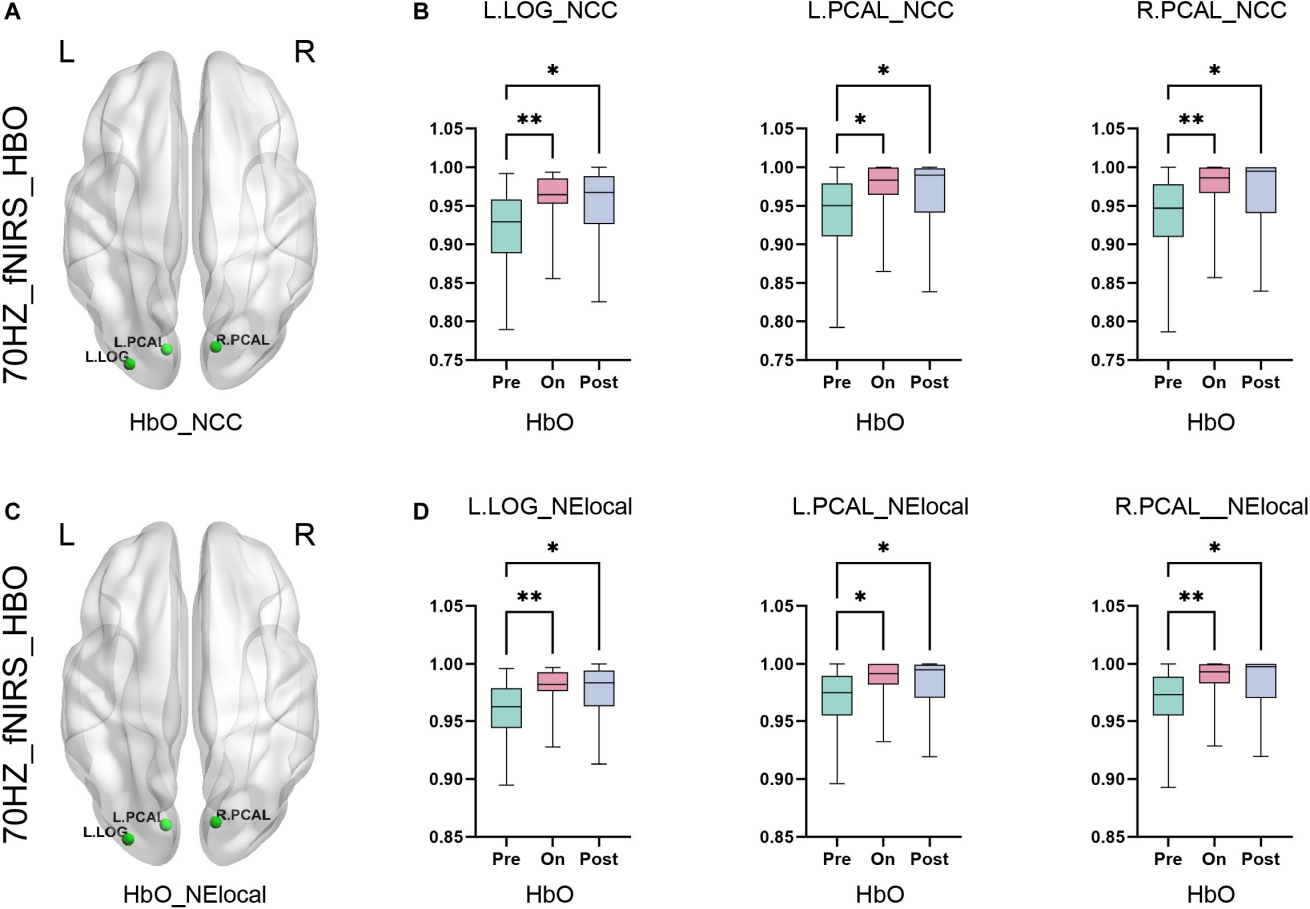
Delayed hemodynamic responses to 70-Hz stimulation in visual cortices. (A) Statistical parametric map displaying regions with elevated NCC for HbO (*P* < 0.05), localized to visual processing cortices (L_LOG, L_PCAL, and R_PCAL). (C) Statistical parametric map displaying regions with elevated NElocal for HbO (*P* < 0.05), localized to visual processing cortices (L_LOG, L_PCAL, and R_PCAL). Both NCC (B) and NElocal (D) exhibited significant increases during the on-stimulation and poststimulation phases relative to the prestimulus baseline. Differential responses localized to the L_LOG, L_PCAL, and R_PCAL. All panels: FDR-corrected; error bars denote SEM; *P < 0.05 and**P < 0.01.

Contrasting with lower frequencies, FC analyses revealed no statistically significant differences in either EEG or fNIRS metrics during 70-, 20-, and 100-Hz stimulation (FDR-corrected, *P* > 0.05). However, node-level analysis at 70 Hz demonstrated frequency-specific hemodynamic alterations. HbO-derived nodal clustering coefficient (NCC) and NElocal exhibited statistically significant increases in the L_lateral occipital (L_LOG), L_pericalcarine (L_PCAL), and R_PCAL during on-stimulation and poststimulation phases relative to baseline (*P* < 0.01) in Fig. 6A and B. Notably, EEG-derived gamma band metrics showed no analogous changes (*P* > 0.05), indicating distinct modality-specific responses. Crucially, the delayed HbO elevation (peak at poststimulation) suggests persistent NVC in visual processing cortices, a finding consistent with prior evidence of SCS-induced metabolic aftereffects in occipital regions.

Five-hertz stimulation elicits immediate electrophysiological responses in frontoparietal networks (NElocal gamma increases in R_Caudal middle frontal [R_CMF]; *P* < 0.05), facilitating real-time consciousness modulation. Seventy-hertz stimulation generates delayed hemodynamic enhancement in visual cortices (HbO-NCC/NElocal in L_LOG/L_PCAL; *P* < 0.05), potentially consolidating arousal through retinotopic pathway recruitment. While previous work established SCS frequency efficacy for DoC [19], our study pioneers anatomically precise biomarkers: 5 Hz targets cingulate motor integration (EEG-NElocal), whereas 70 Hz preferentially engages occipital neurovascular adaptation (fNIRS-NCC/NElocal). This spatiotemporal dissociation may inform personalized neuromodulation protocols.

## Discussion

This study provides the first multimodal characterization of SCS-induced neuromodulation in DoC using EEG–fNIRS derived graph theory metrics. Our findings demonstrate that distinct SCS frequencies (5 and 70 Hz) elicit complementary neurophysiological and hemodynamic responses associated with substantial CRS-R improvements. Previous single-modality fNIRS studies investigating SCS reported increased cerebral blood flow (CBF) and enhanced FC in patients with traumatic brain injury(TBI)-induced DoC during 70-Hz stimulation [17]. Specifically, 70-Hz SCS notably improved FC between prefrontal and occipital regions, indicating frequency-specific modulation of hemodynamic responses and long-range connectivity (with 70 Hz demonstrating superior efficacy). This likely occurs through improved cerebral blood volume and enhanced information transmission via the reticular–thalamocortical pathway [16]. Complementary EEG studies in patients with MCS revealed SCS-mediated augmentation of low-frequency oscillatory activity, particularly in frontal and occipital cortices [41]. Notably, 70-Hz SCS selectively altered relative power and synchrony in frontal delta and gamma bands without significant alterations in other regions [42].

The present study demonstrated that 70-Hz SCS enhanced long-range FC of occipital cortices, a key hub of the consciousness network, while 5-Hz stimulation augmented local gamma-band integration in the R_CMF. These frequency-specific effects align with the established triad of DoC pathophysiology: (a) cerebral hypoperfusion leading to neuronal hypoxia [43]; (b) disrupted thalamocortical circuits [44]; and (c) impaired information integration capacity [45]. The clinical relevance is underscored by SCS’s ability to counteract these pathological processes through quantifiable neurovascular changes, offering a mechanistic basis for its documented therapeutic effects in coma recovery [44,45].

EEG graph metrics revealed distinct arousal mechanisms. Theta-band global efficiency reduction during 5-Hz SCS suggests optimized information transfer, potentially reflecting early restoration of thalamocortical resonance. This is complemented by gamma-band nodal efficiency increases in R_CMF, a region anatomically positioned to integrate motor and cognitive functions [46]. Conversely, 70-Hz SCS induced sustained delta suppression and alpha/beta enhancement, consistent with ABCD-model-aligned recovery of thalamocortical signaling: Depolarized thalamic neurons reinstate tonic firing [12], suppressing pathological slow-wave dominance while augmenting conscious processing-linked alpha activity [45,47,48].

fNIRS confirmed SCS vasoactive properties with regional specificity. Post-5-Hz stimulation, enhanced frontoparietal FC (HbR-based) and nodal changes in left parietal and occipital regions indicate acute reactivation of the dorsal attention network. For 70 Hz, sustained HbO increases in the precuneus and lateral occipital cortex reflect persistent perfusion gains in hubs governing visuospatial integration and self-referential processing [49]. Convergent modulation of left parietal–occipital regions across frequencies validates SCS capacity to restore perfusion in watershed zones vulnerable to hypoperfusion—clinically significant given their metabolic dysfunction in DoC [50].

SCS efficacy arises from synergistic modulation of ascending arousal pathways, neuroplasticity, and hemodynamics: (a) Electrical stimulation activates the reticular–thalamocortical system, facilitating cholinergic excitation of the cortex via basal forebrain projections; (b) frequency-specific neuroplasticity reinforces corticothalamic synapses critical for consciousness maintenance; (c) autonomic modulation reverses cerebral hypoperfusion by inhibiting sympathetic tone while augmenting parasympathetic vasodilation [44,45,51,52]. These mechanisms collectively restore the integrity of the microcircuit model: Normalized thalamocortical drive reactivates prefrontal–striatal systems, enabling functional integration (alpha/beta recovery) and differentiation (gamma nodal changes), thereby targeting the core pathology of impaired information integration. Figure S3 shows a longitudinal comparison of CRS-R scores from baseline to one-month post-activation, demonstrating a statistically significant improvement (*P* < 0.05) that confirms the efficacy of SCS in promoting consciousness recovery. The frequency-dependent effects provide a neurobiological rationale for parameter selection. Five hertz may benefit patients with predominant thalamocortical dysrhythmia (e.g., theta-dominant EEG), while 70 Hz appears optimal for those with metabolic depression in posterior cortices. Future studies should investigate closed-loop SCS systems using real-time EEG–fNIRS feedback to personalize stimulation parameters.

### Limitations and future directions

While our study provides novel insights into the immediate neurophysiological modulation induced by SCS in patients with DoC, we acknowledge several limitations inherent to the study design. First, the sample size, although consistent with or exceeding similar invasive neuromodulation studies in this rare clinical population, limits the statistical power for broad generalization [53–55]. To address the potential reliability concerns inherent in small-cohort studies, we used rigorous statistical corrections (FDR and Bonferroni) to control the family-wise error rate, ensuring that the reported frequency-specific network reconfigurations are robust rather than stochastic artifacts.

Randomized controlled trials represent the gold standard for validating therapeutic efficacy; however, it is imperative to distinguish the primary objective of this study—namely, the identification of acute mechanistic biomarkers of responsiveness—from large-scale clinical efficacy trials [56,57]. This work serves as a pioneering translational exploration, utilizing integrated EEG–fNIRS to elucidate the immediate neurophysiological underpinnings of SCS [58]. By adopting a stringent within-subject, prestimulation versus poststimulation paradigm, we utilized each patient as their own internal control [59]. This design effectively mitigates the inherent intersubject variability characteristic of DoC pathology and enhances the statistical sensitivity required to detect acute, transient modulations in neural dynamics within a relatively compact cohort. The immediate reconfigurations observed in frontoparietal connectivity and spectral power provide “proof-of-concept” evidence for the biological engagement of target neural circuits by SCS [60].

We acknowledge that while our pilot cohort (*n* = 16) provides sufficient power for characterizing these acute mechanistic signatures, it lacks the stratification capacity to account for heterogeneous etiologies (e.g., TBI versus hypoxic-ischemic encephalopathy [HIE]), where varying degrees of thalamocortical tract integrity may differentially modulate SCS responsiveness [61]. To address the limitations inherent in this early-phase investigation, our team is currently conducting a registered, multicenter longitudinal trial. This ongoing study is explicitly designed to compare SCS against a sham–control group, aiming to provide definitive evidence regarding therapeutic superiority and sustained consciousness recovery. The neurophysiological biomarkers identified in the present study will serve as the foundational framework for this subsequent trial, transitioning the field from empirical neuromodulation toward a model of precision, closed-loop network therapeutics.

Our network analysis faces inherent technical and methodological constraints that warrant consideration. Standard EEG connectivity analyses cannot resolve high-frequency gamma synchrony (>80 Hz) implicated in conscious integration [2], while hemodynamic delays in fNIRS restrict temporal precision for dynamic FC assessments [62]. A central limitation of applying EEG source localization techniques, such as wMNE and sLORETA, to patients with DoC stems from the fundamental ill-posedness of the inverse problem and the inherent solution bias toward superficial sources, a challenge that is critically exacerbated when using standardized template head models (e.g., Colin27) [63]. Mathematically, the accuracy of source reconstruction is contingent upon a precise forward model that incorporates individual anatomical geometry, tissue conductivities, and pathology-7. In patients with DoC and severe brain injuries, the use of a generic template fails to account for patient-specific alterations such as focal lesions, diffuse atrophy, and ventricular enlargement [64,65]. This mismatch systematically distorts the leadfield matrix, introducing localization errors that are not merely random noise but structured biases [66]. Consequently, estimated neural generators may be erroneously displaced toward the (intact) cortical surface or away from lesioned areas, potentially misrepresenting critical pathophysiological signatures, such as the integrity of deep thalamocortical circuits or perilesional network dynamics that are prognostically relevant [67]. Future advancements must prioritize the development and validation of lesion-informed, individualized forward models, potentially integrated with multimodal imaging constraints, to mitigate these systematic errors and enhance the neuroanatomical validity of source-space connectivity analyses in this vulnerable patient population [68].

## Conclusion

This integrated EEG–fNIRS study resolves a fundamental paradox in SCS for DoC by establishing a frequency-dependent duality of neuromodulator mechanisms. We demonstrate that 5-Hz SCS acutely enhances front limbic engagement through significant theta-band global efficiency elevation (*P* < 0.01) coupled with gamma-band nodal efficiency surges in the cingulate motor area, indicating preferential electrophysiological facilitation without concomitant hemodynamic changes—a phenomenon termed neurovascular decoupling. Conversely, 70-Hz stimulation selectively recruits retinotopic pathways via delayed hemodynamic responses in visual cortices (*P* < 0.01) devoid of EEG correlates, revealing frequency-specific vascular recruitment. This mechanistic dissociation provides the first neurophysiological rationale for optimizing SCS protocols: 5 Hz targets preserved front limbic circuits for rapid arousal modulation, whereas 70 Hz engages compromised visual pathways for sustained metabolic recovery. Critically, our graph-theoretical biomarkers, theta-band global efficiency and occipital hemodynamic responses, transcend behavioral scales by offering quantifiable, modality-specific indices for treatment personalization. These findings inaugurate a paradigm shift toward precision network therapeutics, where closed-loop systems could dynamically titrate stimulation parameters against real-time nodal efficiency metrics. Future validation in larger cohorts should integrate tractography stratification to optimize electrode placement, ultimately advancing SCS from empirical application toward mechanism-guided neuromodulation in DoC states.

## Data Availability

All data reported in this paper will be shared by the lead contact upon request. Any additional information required to reanalyze the data reported in this paper is available from the lead contact upon request.
