# Supplementary material for: Graph-Theoretical Signature from Neural and Vascular Signals Reveals Spinal Cord Stimulation Frequency-Specific Brain Network in Disorders of Consciousness Patients
Source: Cyborg Bionic Syst. 2026 Apr 23;7:0539. doi: 10.34133/cbsystems.0539 (PMC13103464; doi:10.34133/cbsystems.0539)
Supplement: Supplementary 1 — Figs. S1 to S3 Table S1 [file cbsystems.0539.f1.zip › Supplementary Manuscript.docx]

**Supplemental Information**

Graph-Theoretical Signature from Neural and Vascular Signals Reveal Spinal Cord Stimulation Frequency-Specific Brain Network in Disorders of Consciousness Patients

Nan Wang^1,2#^, Xiaoke Chai^1,3#^, Yifang He^4^, Jiuxiang Song^5^, Tianqing Cao^6^, Qiheng He^1^, Sipeng Zhu^1,^ Yitong Jia^1,^ Juanning Si^4^, Yi Yang^1,3,7^*, Jizong Zhao^1^*

1Department of Neurosurgery, Beijing Tiantan Hospital, Capital Medical University, Beijing, China

2Department of Neurosurgery, Peking Union Medical College Hospital, Chinese Academy of Medical Sciences and Peking Union Medical College, Beijing, China

3China National Clinical Research Center for Neurological Diseases, Beijing, China

4School of Instrumentation Science and Opto-Electronics Engineering, Beijing Information Science and Technology University, Beijing, China

5School of Advanced Manufacturing, Nanchang University, Nanchang, Jiangxi, China

6Department of Neurosurgery, Aviation General Hospital, Beijing, China

7Brain Computer Interface Transitional Research Center, Beijing Tiantan Hospital, Capital Medical University, Beijing, China

**† These authors have contributed equally to this work.**

*** Correspondence:**

*Jizong Zhao, Department of Neurosurgery, Beijing Tiantan Hospital, Capital Medical University, No.119, South Fourth Ring Road, Fengtai District, Beijing, 100070, China. E-mail: zhaojizong@bjtth.org

*Yi Yang, Department of Neurosurgery, Beijing Tiantan Hospital, Capital Medical University, No.119, South Fourth Ring Road, Fengtai District, Beijing, 100070, China. E-mail: yangyi_81nk@163.com.

**MATERIALS AND METHODS**

**Brain Network Analysis Based on Graph Theory**

**Small World Index (SWI)**

The small-world network analysis was subsequently conducted on the constructed binary matrices. The clustering coefficient (CC) and characteristic path length (CPL) of the real network were calculated. The CC measures the degree to which nodes in the network tend to cluster together, while the CPL reflects the average shortest path between any two nodes in the network. To determine the small-world properties, 100 random networks with the same number of nodes and edges as the real network were generated. The average clustering coefficient $C_{\mathrm{rand}}$and average shortest path length ($L_{\mathrm{rand}}$) of these 100 random networks were calculated.

As defined, the ratio (${\gamma=C}_{\mathrm{real}}/C_{\mathrm{rand}} , \gamma>1$), where $C_{\mathrm{real}}$ is the clustering coefficient of the real network. When $\lambda>1$, it indicates that the real network has a higher clustering tendency compared to random networks. The ratio ${\gamma=C}_{\mathrm{real}}/C_{\mathrm{rand}}$, with $L_{\mathrm{real}}$ being the CPL of the real network. When $\lambda\approx1$, the CPL of the real network is like that of random networks. The small-world property $\sigma$ is calculated as $\sigma=\gamma/\lambda$. If $\sigma>1,$ the network is considered to possess small-world properties, which suggests an optimized balance between local integration and global information transfer in the brain network during SCS treatment for DoCs.

**(Nodal) Clustering Coefficient ((N)CC,)**

The CC of the network is the average of the clustering coefficients of all nodes. The CC is defined as the ratio of the number of edges between "other nodes" directly connected to node i within the network to the maximum possible number of edges between these "other nodes".

The formula is as follows:

$$C_{i}=\frac{E_{i}}{\frac{1}{2}k_{i}(k_{i}-1)}$$

$$C=\frac{1}{N}\sum_{i} C_{i}$$

Where $E_{i}$ denotes the number of edges between "other nodes" directly connected to node i, and $k_{i}$ denotes the degree of connectivity of node i. $\frac{1}{2}k_{i}(k_{i}-1)$ is the number of connected edges between "other nodes" if they are connected in pairs. The node attribute $C_{i}$ measures the degree of agglomeration of the node, and the global attribute $C_{i}$ measures the degree of agglomeration of the network.

**Global Efficiency (Eglobal)**

Eglobal is defined as the average reciprocal of the shortest path length between all node pairs in the whole-brain network. It measures the efficiency of information transmission in the network. The higher the Eglobal, the higher the overall efficiency of information transmission in the network.

The formula is as follows:

$$S=\frac{1}{N(N-1)}\sum_{i,j,i\neq j} \frac{1}{d_{\mathrm{ij}}}$$

Where N is the number of nodes and $d_{ij}$ represents the distance between node i and node j.

**Nodal Global Efficiency (NEglobal)**

The global efficiency of node i is defined as:

$$E_{\mathrm{nodal}}(i)=\frac{1}{N-1}\sum_{j,j\neq i} \frac{1}{d_{\mathrm{ij}}}$$

Where $d_{ij}$represents the shortest path between node i and node j, reflecting the ability of the node to transmit information within the network. The larger $E_{nodal}(i)$ is the faster the information transmission between that node and other nodes.

**Nodal Local Efficiency (NElocal)**

Like the Eglobal of node i, the NElocal of node i measures the compactness of the small network composed of the neighboring nodes of node i.

The formula is as follows:

$${E_{\mathrm{local}}(i)=E}_{\mathrm{global}}\left( G_{i} \right)=\frac{1}{N_{G_{i}}(N_{G_{i}}-1)}\sum_{j,k,j\neq k,j\in G_{i},k\in G_{i}} \frac{1}{d_{\mathrm{jk}}}$$

Where $G_{i}$ is the subnetwork composed of “other nodes” directly connected to node i, $N_{G_{i}}$is the total number of nodes in the subnetwork $G_{i}$, and $d_{ij}$ represents the shortest path between node i and node j.

**Degree Centrality (DC)**

DC is defined as the number of nodes directly connected to a given node or the number of edges on that node. DC measures the closeness of a single node's direct connections to other nodes in a network. The degree value of a brain region reflects the number of connections between that region and other brain regions. A brain region with a high degree value typically indicates that it has a high degree of connectivity in the brain network and may play a more important role, such as information integration or transmission.

**Betweenness Centrality (BC)**

Intermediary centrality is defined as the frequency (proportion) with which a node appears in the shortest paths between all other nodes. It measures the extent to which a node serves as a bridge, intermediary, or hub within a network, reflecting how critically positioned it is along the key paths for information transmission between other nodes. The higher a node's intermediary centrality, the more significant its role in information transmission within the network.

The formula is as follows:

$$\mathrm{BC}_{i}=\sum_{j,k,j\neq k\neq i} \frac{\sigma_{\mathrm{jk}}(i)}{\sigma_{\mathrm{jk}}}$$

Where $\sigma_{jk}$ represents the number of shortest paths between node j and node k, and $\sigma_{jk}\left( i \right)$ denotes the number of paths between node j and node k that pass-through node i.

**RESULTS**

**Frequency-Specific Modulation of Global Network Topology**

**
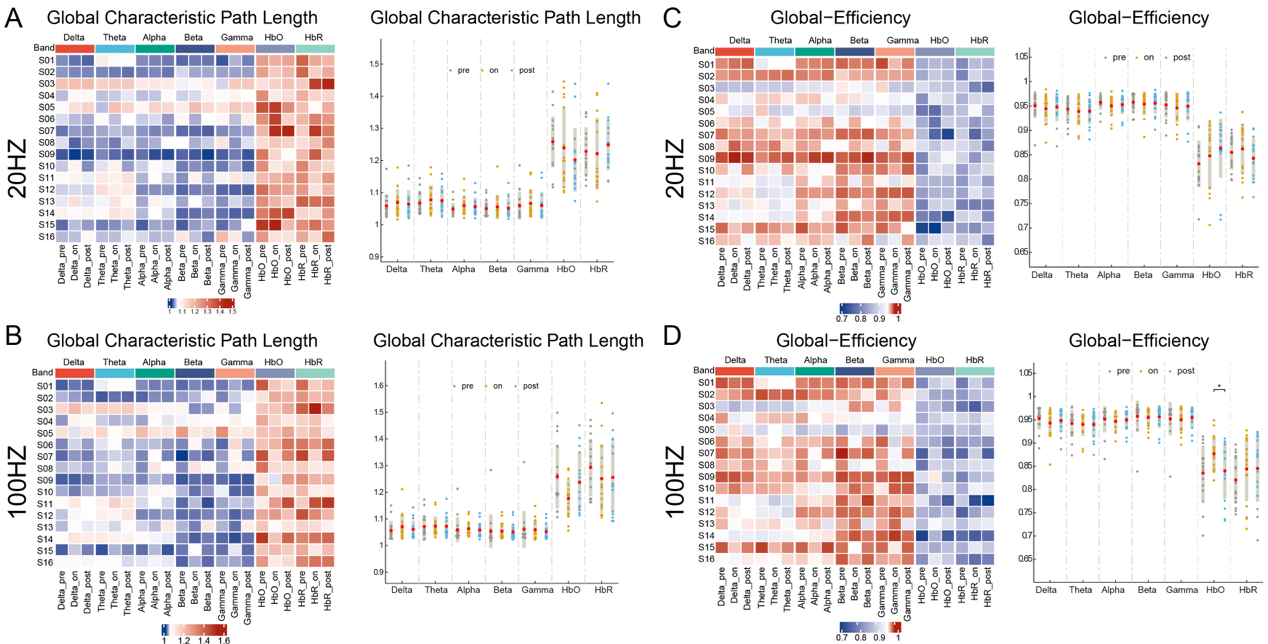
**

**Supplementary Fig. 1**. **Frequency-insensitive network responses at 20 and 100 Hz stimulation.** **(A)** CPL dynamics during 20 Hz stimulation. Individual-subject heatmaps display pre-/on-/post-stimulus values for EEG bands (delta, theta, alpha, beta, gamma) and fNIRS hemodynamics (ΔHbO, ΔHbR); group-averaged statistical plots confirm no significant alterations (FDR-corrected; *p > 0.05*). **(B)** Global efficiency during 20 Hz stimulation. Individual heatmaps show subject-specific trajectories while group analysis demonstrates the absence of modulation across all metrics. **(C)** CPL reorganization during 100 Hz stimulation. Individual-subject heatmaps reveal variability; group statistics show no EEG band changes. **(D)** Global efficiency modulation during 100 Hz stimulation. Individual trajectories contrast with group-averaged results exhibiting selective ΔHbO reduction post-stimulation (post vs. on, *p < 0.05*).

**Consciousness recovery following SCS**

Longitudinal assessment using the CRS-R served as the primary objective metric to evaluate the efficacy of SCS in promoting consciousness recovery within our cohort. Critically, and aligning with established benchmarks for clinically meaningful neurological improvement [1, 2], an increase in CRS-R score of > 3 points was observed in responsive patients, confirming the transition towards a higher level of awareness. **Supplementary Figure 2** graphically depicts the comparative analysis of CRS-R scores recorded at baseline (pre-implantation) and at the one-month post-activation time point. A statistically significant augmentation in CRS-R scores was evident following one month of continuous SCS therapy (*p < 0.05*). This pronounced elevation underscores the neuromodulatory capacity of SCS to facilitate significant functional gains in consciousness levels within this critical post-intervention window. Collectively, the quantifiable CRS-R progression exceeding the validated threshold of 3 points, coupled with the significant statistical outcome, provides robust objective evidence for the consciousness-promoting effect achieved through our SCS intervention protocol.


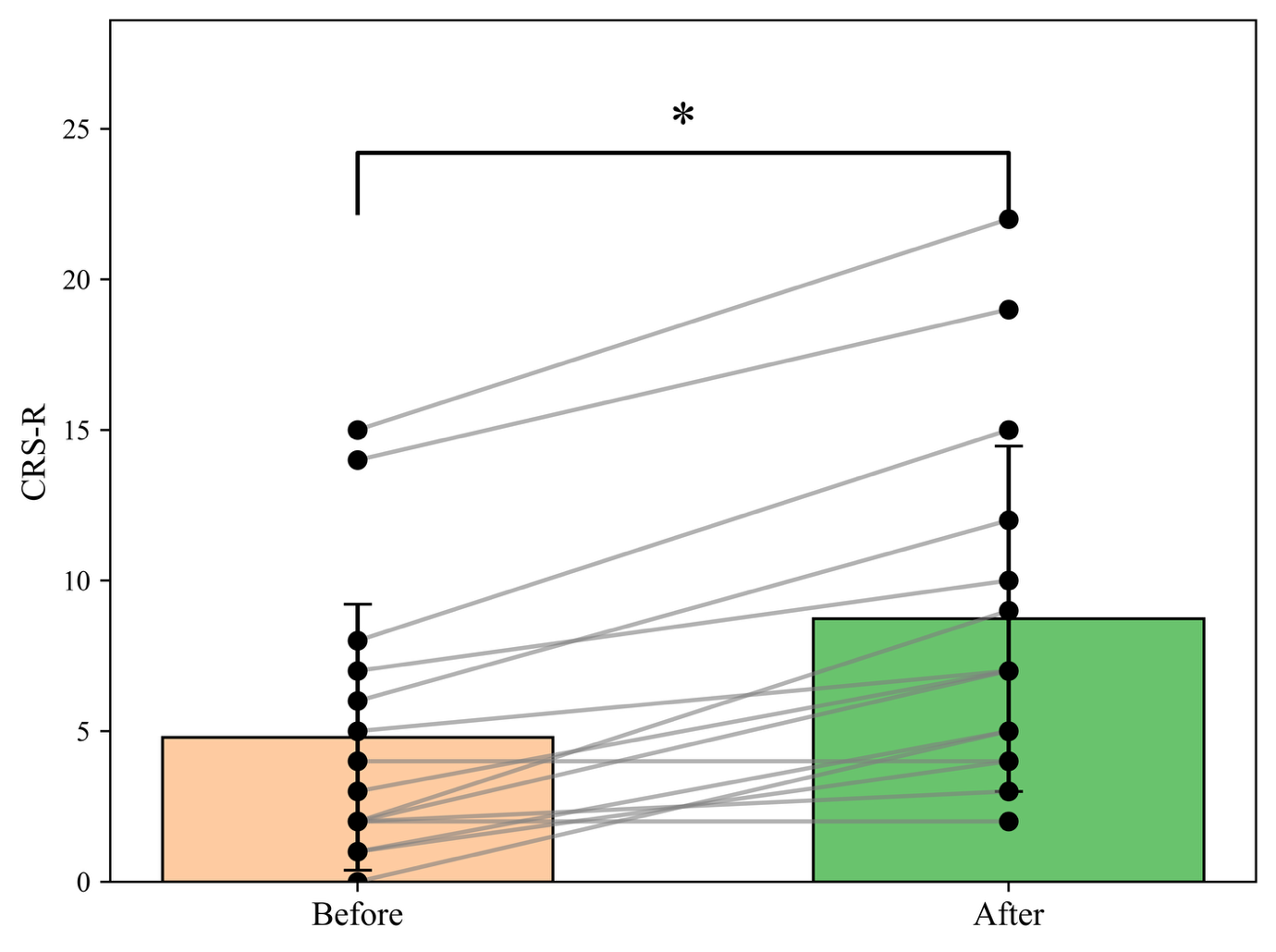


**Supplementary Fig. 2. Significant Improvement in CRS-R Scores Following SCS.** Bar graph depicting mean CRS-R total scores (± SEM/SD) at baseline (pre-treatment) and after 1 month of continuous SCS therapy (n = 16). Post-treatment scores demonstrated a statistically significant increase compared to baseline (paired t-test； *p* < 0.05). The magnitude of improvement exceeded the clinically established threshold of > 3 points, indicating meaningful recovery of consciousness. **p < 0.05*.

References

1. Seel, R.T., et al., *Assessment scales for disorders of consciousness: evidence-based recommendations for clinical practice and research.* Arch Phys Med Rehabil, 2010. **91**(12): p. 1795-813.

2. Giacino, J.T., K. Kalmar, and J. Whyte, *The JFK Coma Recovery Scale-Revised: measurement characteristics and diagnostic utility.* Arch Phys Med Rehabil, 2004. **85**(12): p. 2020-9.
