# Supplementary material for: Graph-Theoretical Signature from Neural and Vascular Signals Reveals Spinal Cord Stimulation Frequency-Specific Brain Network in Disorders of Consciousness Patients
Source: Cyborg Bionic Syst. 2026 Apr 23;7:0539. doi: 10.34133/cbsystems.0539 (PMC13103464; doi:10.34133/cbsystems.0539)
Supplement: Supplementary 1 — Figs. S1 to S3 Table S1 [file cbsystems.0539.f1.zip › Supplementary Table 1.docx]

**Supplementary Table 1. Summary of EEG data preprocessing quality metrics for each participant**

| Idx | Filename | TotalDuration | RetainDuration | nBadCh | nRetainIC |
| --- | --- | --- | --- | --- | --- |
| 1 | sub01_5hz_pre | 115.3242188 | 98 | 2 | 21 |
| 2 | sub01_5hz_on | 558.9921875 | 458 | 0 | 18 |
| 3 | sub01_5hz_post | 855.796875 | 758 | 0 | 22 |
| 4 | sub01_70hz_pre | 118.0039063 | 86 | 1 | 21 |
| 5 | sub01_70hz_on | 503.2773438 | 416 | 1 | 22 |
| 6 | sub01_70hz_post | 1091.242188 | 948 | 1 | 16 |
| 7 | sub01_20hz_pre | 117.6640625 | 98 | 1 | 22 |
| 8 | sub01_20hz_on | 580.9101563 | 466 | 1 | 21 |
| 9 | sub01_20hz_post | 931.3203125 | 836 | 0 | 17 |
| 10 | sub01_10hz_pre | 115.3203125 | 102 | 2 | 16 |
| 11 | sub01_10hz_on | 595.6796875 | 512 | 0 | 14 |
| 12 | sub01_10hz_post | 939.046875 | 734 | 0 | 19 |
| 13 | sub02_5hz_pre | 120.0039063 | 94 | 0 | 24 |
| 14 | sub02_5hz_on | 593.3203125 | 492 | 0 | 16 |
| 15 | sub02_5hz_post | 910 | 824 | 1 | 19 |
| 16 | sub02_70hz_pre | 116.6640625 | 94 | 1 | 24 |
| 17 | sub02_70hz_on | 597.3359375 | 532 | 0 | 21 |
| 18 | sub02_70hz_post | 907.3203125 | 802 | 0 | 22 |
| 19 | sub02_20hz_pre | 119.0039063 | 104 | 0 | 21 |
| 20 | sub02_20hz_on | 598.0195313 | 526 | 0 | 22 |
| 21 | sub02_20hz_post | 901.9804688 | 776 | 0 | 23 |
| 22 | sub02_10hz_pre | 115.3242188 | 96 | 0 | 23 |
| 23 | sub02_10hz_on | 599.0195313 | 556 | 0 | 22 |
| 24 | sub02_10hz_post | 889.3203125 | 802 | 0 | 19 |
| 25 | sub03_5hz_pre | 117.3242188 | 102 | 0 | 23 |
| 26 | sub03_5hz_on | 597 | 488 | 0 | 25 |
| 27 | sub03_5hz_post | 623 | 516 | 0 | 25 |
| 28 | sub03_70hz_pre | 120.0039063 | 98 | 0 | 28 |
| 29 | sub03_70hz_on | 600.0195313 | 486 | 0 | 27 |
| 30 | sub03_70hz_post | 571.6601563 | 480 | 0 | 24 |
| 31 | sub03_20hz_pre | 120.0039063 | 100 | 0 | 25 |
| 32 | sub03_20hz_on | 600.0195313 | 484 | 0 | 26 |
| 33 | sub03_20hz_post | 982 | 792 | 0 | 25 |
| 34 | sub03_10hz_pre | 120.0039063 | 104 | 0 | 25 |
| 35 | sub03_10hz_on | 600.0195313 | 496 | 0 | 23 |
| 36 | sub03_10hz_post | 866 | 750 | 0 | 22 |
| 37 | sub04_5hz_pre | 116.984375 | 100 | 0 | 20 |
| 38 | sub04_5hz_on | 596.3203125 | 546 | 0 | 16 |
| 39 | sub04_5hz_post | 943 | 896 | 1 | 13 |
| 40 | sub04_70hz_pre | 120.0039063 | 112 | 1 | 19 |
| 41 | sub04_70hz_on | 600.0195313 | 550 | 0 | 15 |
| 42 | sub04_70hz_post | 910 | 830 | 0 | 16 |
| 43 | sub04_20hz_pre | 120.0039063 | 112 | 0 | 18 |
| 44 | sub04_20hz_on | 593.5820313 | 552 | 0 | 14 |
| 45 | sub04_20hz_post | 928 | 868 | 0 | 15 |
| 46 | sub04_10hz_pre | 120.0039063 | 118 | 0 | 19 |
| 47 | sub04_10hz_on | 598 | 544 | 0 | 14 |
| 48 | sub04_10hz_post | 943 | 876 | 0 | 16 |
| 49 | sub05_5hz_pre | 92.0078125 | 76 | 2 | 9 |
| 50 | sub05_5hz_on | 505.890625 | 406 | 5 | 5 |
| 51 | sub05_5hz_post | 704.890625 | 578 | 6 | 12 |
| 52 | sub05_70hz_pre | 108.265625 | 80 | 5 | 13 |
| 53 | sub05_70hz_on | 478.6835938 | 374 | 4 | 11 |
| 54 | sub05_70hz_post | 838.7265625 | 722 | 4 | 13 |
| 55 | sub05_20hz_pre | 120.0039063 | 114 | 2 | 14 |
| 56 | sub05_20hz_on | 580.8945313 | 526 | 3 | 5 |
| 57 | sub05_20hz_post | 770.2734375 | 618 | 3 | 12 |
| 58 | sub05_10hz_pre | 113.2851563 | 92 | 6 | 8 |
| 59 | sub05_10hz_on | 572.15625 | 486 | 3 | 9 |
| 60 | sub05_10hz_post | 886.375 | 820 | 0 | 7 |
| 61 | sub06_5hz_pre | 101.484375 | 90 | 0 | 20 |
| 62 | sub06_5hz_on | 524.0546875 | 414 | 0 | 17 |
| 63 | sub06_5hz_post | 874.1445313 | 762 | 2 | 15 |
| 64 | sub06_70hz_pre | 116.6445313 | 74 | 1 | 18 |
| 65 | sub06_70hz_on | 537.8320313 | 422 | 1 | 18 |
| 66 | sub06_70hz_post | 825.6484375 | 696 | 2 | 19 |
| 67 | sub06_20hz_pre | 116.6640625 | 98 | 1 | 18 |
| 68 | sub06_20hz_on | 538.8398438 | 432 | 0 | 16 |
| 69 | sub06_20hz_post | 920 | 770 | 1 | 19 |
| 70 | sub06_10hz_pre | 103.5625 | 72 | 3 | 22 |
| 71 | sub06_10hz_on | 585.2773438 | 492 | 1 | 18 |
| 72 | sub06_10hz_post | 901.8789063 | 752 | 1 | 17 |
| 73 | sub07_5hz_pre | 117.3242188 | 102 | 0 | 26 |
| 74 | sub07_5hz_on | 590.9609375 | 554 | 0 | 20 |
| 75 | sub07_5hz_post | 930.3203125 | 870 | 0 | 22 |
| 76 | sub07_70hz_pre | 119.0039063 | 102 | 1 | 21 |
| 77 | sub07_70hz_on | 597 | 526 | 1 | 23 |
| 78 | sub07_70hz_post | 919.5351563 | 824 | 1 | 20 |
| 79 | sub07_20hz_pre | 119.0039063 | 110 | 1 | 23 |
| 80 | sub07_20hz_on | 590.9609375 | 506 | 0 | 25 |
| 81 | sub07_20hz_post | 923.9609375 | 854 | 1 | 26 |
| 82 | sub07_10hz_pre | 118.6640625 | 108 | 1 | 26 |
| 83 | sub07_10hz_on | 600.0195313 | 530 | 1 | 22 |
| 84 | sub07_10hz_post | 1017 | 942 | 0 | 21 |
| 85 | sub08_5hz_pre | 116.3007813 | 88 | 2 | 16 |
| 86 | sub08_5hz_on | 600.0195313 | 546 | 2 | 18 |
| 87 | sub08_5hz_post | 926.1601563 | 778 | 1 | 22 |
| 88 | sub08_70hz_pre | 112.984375 | 82 | 2 | 20 |
| 89 | sub08_70hz_on | 526.2734375 | 402 | 1 | 18 |
| 90 | sub08_70hz_post | 907.6210938 | 782 | 2 | 15 |
| 91 | sub08_20hz_pre | 118.3203125 | 110 | 2 | 16 |
| 92 | sub08_20hz_on | 573.078125 | 488 | 2 | 18 |
| 93 | sub08_20hz_post | 912.21875 | 784 | 0 | 18 |
| 94 | sub08_10hz_pre | 118.6640625 | 106 | 2 | 17 |
| 95 | sub08_10hz_on | 558.7578125 | 436 | 1 | 15 |
| 96 | sub08_10hz_post | 646.8632813 | 522 | 3 | 15 |
| 97 | sub09_5hz_pre | 115.6210938 | 104 | 1 | 21 |
| 98 | sub09_5hz_on | 587.921875 | 516 | 2 | 18 |
| 99 | sub09_5hz_post | 1042.878906 | 956 | 0 | 17 |
| 100 | sub09_70hz_pre | 116.3242188 | 108 | 2 | 16 |
| 101 | sub09_70hz_on | 575.515625 | 474 | 0 | 17 |
| 102 | sub09_70hz_post | 873.859375 | 706 | 0 | 18 |
| 103 | sub09_20hz_pre | 114.3046875 | 100 | 1 | 20 |
| 104 | sub09_20hz_on | 579.1523438 | 468 | 0 | 20 |
| 105 | sub09_20hz_post | 892.28125 | 750 | 0 | 20 |
| 106 | sub09_10hz_pre | 110.265625 | 90 | 0 | 22 |
| 107 | sub09_10hz_on | 582.6210938 | 478 | 0 | 21 |
| 108 | sub09_10hz_post | 895.4335938 | 832 | 0 | 20 |
| 109 | sub10_5hz_pre | 120.0039063 | 108 | 1 | 17 |
| 110 | sub10_5hz_on | 595.3203125 | 536 | 1 | 20 |
| 111 | sub10_5hz_post | 895.3203125 | 840 | 1 | 16 |
| 112 | sub10_70hz_pre | 120.0039063 | 116 | 1 | 21 |
| 113 | sub10_70hz_on | 600.0195313 | 548 | 1 | 20 |
| 114 | sub10_70hz_post | 876.0429688 | 770 | 1 | 17 |
| 115 | sub10_20hz_pre | 116.984375 | 106 | 1 | 22 |
| 116 | sub10_20hz_on | 585.5585938 | 492 | 1 | 17 |
| 117 | sub10_20hz_post | 919.9804688 | 850 | 1 | 19 |
| 118 | sub10_10hz_pre | 120.0039063 | 118 | 1 | 21 |
| 119 | sub10_10hz_on | 589.3007813 | 508 | 1 | 21 |
| 120 | sub10_10hz_post | 893.1132813 | 782 | 1 | 20 |
| 121 | sub11_5hz_pre | 113.625 | 100 | 0 | 24 |
| 122 | sub11_5hz_on | 592.9804688 | 528 | 0 | 22 |
| 123 | sub11_5hz_post | 984.9804688 | 910 | 0 | 23 |
| 124 | sub11_70hz_pre | 118.6640625 | 108 | 0 | 25 |
| 125 | sub11_70hz_on | 593.9804688 | 518 | 0 | 23 |
| 126 | sub11_70hz_post | 996 | 936 | 1 | 22 |
| 127 | sub11_20hz_pre | 116.984375 | 100 | 0 | 23 |
| 128 | sub11_20hz_on | 539.4882813 | 456 | 1 | 23 |
| 129 | sub11_20hz_post | 1213.683594 | 1094 | 0 | 17 |
| 130 | sub11_10hz_pre | 118.6640625 | 98 | 1 | 25 |
| 131 | sub11_10hz_on | 600.0195313 | 556 | 2 | 21 |
| 132 | sub11_10hz_post | 1005 | 890 | 1 | 23 |
| 133 | sub12_5hz_pre | 120.0039063 | 106 | 0 | 27 |
| 134 | sub12_5hz_on | 592.3007813 | 500 | 0 | 26 |
| 135 | sub12_5hz_post | 920 | 812 | 0 | 27 |
| 136 | sub12_70hz_pre | 120.0039063 | 102 | 0 | 29 |
| 137 | sub12_70hz_on | 598.3359375 | 498 | 0 | 27 |
| 138 | sub12_70hz_post | 921 | 820 | 0 | 24 |
| 139 | sub12_20hz_pre | 120.0039063 | 108 | 0 | 30 |
| 140 | sub12_20hz_on | 600.0195313 | 516 | 0 | 30 |
| 141 | sub12_20hz_post | 907.5546875 | 776 | 0 | 25 |
| 142 | sub12_10hz_pre | 120.0039063 | 100 | 0 | 29 |
| 143 | sub12_10hz_on | 600.0195313 | 544 | 1 | 25 |
| 144 | sub12_10hz_post | 954.6601563 | 822 | 0 | 28 |
| 145 | sub13_5hz_pre | 105.2382813 | 82 | 9 | 11 |
| 146 | sub13_5hz_on | 582.4804688 | 500 | 7 | 4 |
| 147 | sub13_5hz_post | 779.578125 | 532 | 5 | 10 |
| 148 | sub13_70hz_pre | 106.5625 | 84 | 4 | 10 |
| 149 | sub13_70hz_on | 365.0273438 | 184 | 7 | 13 |
| 150 | sub13_70hz_post | 1563.921875 | 1194 | 3 | 11 |
| 151 | sub13_20hz_pre | 106.5273438 | 90 | 4 | 11 |
| 152 | sub13_20hz_on | 585.2382813 | 520 | 5 | 10 |
| 153 | sub13_20hz_post | 884.234375 | 650 | 6 | 12 |
| 154 | sub13_10hz_pre | 111.28125 | 90 | 7 | 12 |
| 155 | sub13_10hz_on | 356.2460938 | 274 | 6 | 13 |
| 156 | sub13_10hz_post | 800.9570313 | 650 | 8 | 15 |
| 157 | sub14_5hz_pre | 120.0039063 | 100 | 0 | 22 |
| 158 | sub14_5hz_on | 596.3398438 | 516 | 0 | 19 |
| 159 | sub14_5hz_post | 886.4648438 | 664 | 0 | 25 |
| 160 | sub14_70hz_pre | 109.2421875 | 68 | 0 | 22 |
| 161 | sub14_70hz_on | 588.21875 | 412 | 0 | 24 |
| 162 | sub14_70hz_post | 949.6015625 | 712 | 0 | 23 |
| 163 | sub14_20hz_pre | 116.3242188 | 74 | 0 | 22 |
| 164 | sub14_20hz_on | 565.0664063 | 396 | 0 | 21 |
| 165 | sub14_20hz_post | 947.4453125 | 700 | 0 | 21 |
| 166 | sub14_10hz_pre | 114.9648438 | 90 | 0 | 20 |
| 167 | sub14_10hz_on | 590.9609375 | 490 | 0 | 20 |
| 168 | sub14_10hz_post | 907.9414063 | 682 | 0 | 22 |
| 169 | sub15_5hz_pre | 105.5859375 | 60 | 0 | 15 |
| 170 | sub15_5hz_on | 588.9804688 | 524 | 0 | 15 |
| 171 | sub15_5hz_post | 905.8632813 | 814 | 0 | 15 |
| 172 | sub15_70hz_pre | 104.203125 | 72 | 0 | 19 |
| 173 | sub15_70hz_on | 584.6015625 | 504 | 0 | 14 |
| 174 | sub15_70hz_post | 802.3398438 | 536 | 0 | 16 |
| 175 | sub15_20hz_pre | 120.0039063 | 102 | 0 | 15 |
| 176 | sub15_20hz_on | 553.3632813 | 418 | 0 | 14 |
| 177 | sub15_20hz_post | 941.9804688 | 836 | 0 | 16 |
| 178 | sub15_10hz_pre | 89.7890625 | 46 | 0 | 15 |
| 179 | sub15_10hz_on | 568.1640625 | 486 | 0 | 10 |
| 180 | sub15_10hz_post | 851.9375 | 608 | 1 | 18 |
| 181 | sub16_5hz_pre | 120.0039063 | 94 | 0 | 18 |
| 182 | sub16_5hz_on | 597.3398438 | 468 | 1 | 18 |
| 183 | sub16_5hz_post | 999.3203125 | 810 | 0 | 21 |
| 184 | sub16_70hz_pre | 117.3242188 | 86 | 0 | 18 |
| 185 | sub16_70hz_on | 598.6796875 | 450 | 0 | 22 |
| 186 | sub16_70hz_post | 917 | 650 | 0 | 22 |
| 187 | sub16_20hz_pre | 118.6640625 | 90 | 0 | 25 |
| 188 | sub16_20hz_on | 556.5625 | 412 | 3 | 24 |
| 189 | sub16_20hz_post | 902.9804688 | 676 | 0 | 24 |
| 190 | sub16_10hz_pre | 117.6640625 | 82 | 0 | 23 |
| 191 | sub16_10hz_on | 600.0195313 | 432 | 1 | 22 |
| 192 | sub16_10hz_post | 935 | 660 | 0 | 26 |
