# Supplementary figures and images for: Graph-Theoretical Signature from Neural and Vascular Signals Reveals Spinal Cord Stimulation Frequency-Specific Brain Network in Disorders of Consciousness Patients
Source: Cyborg Bionic Syst. 2026 Apr 23;7:0539. doi: 10.34133/cbsystems.0539 (PMC13103464; doi:10.34133/cbsystems.0539)

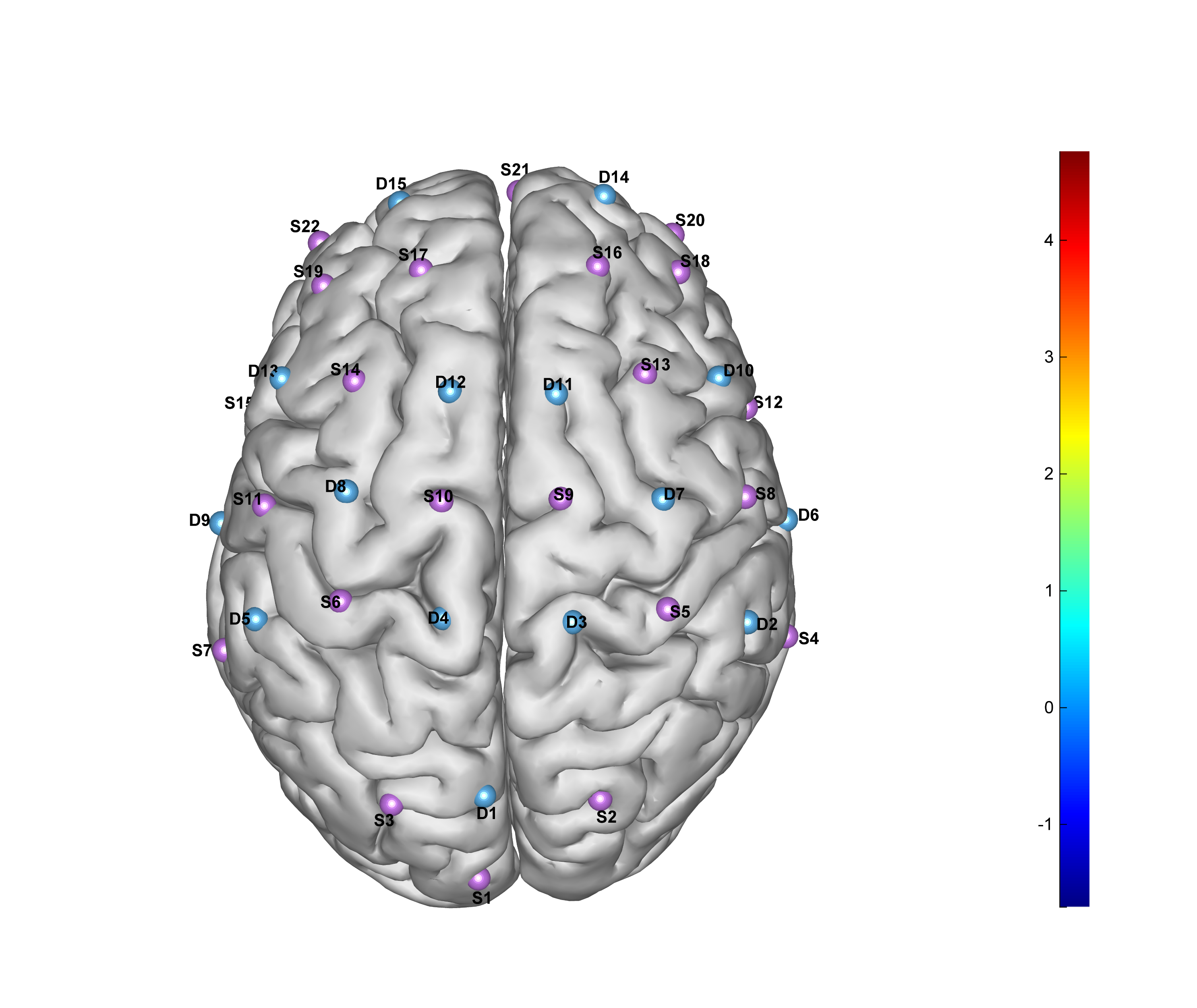

Supplement: Supplementary 1 — Figs. S1 to S3 Table S1 [file cbsystems.0539.f1.zip › S1 .tif]

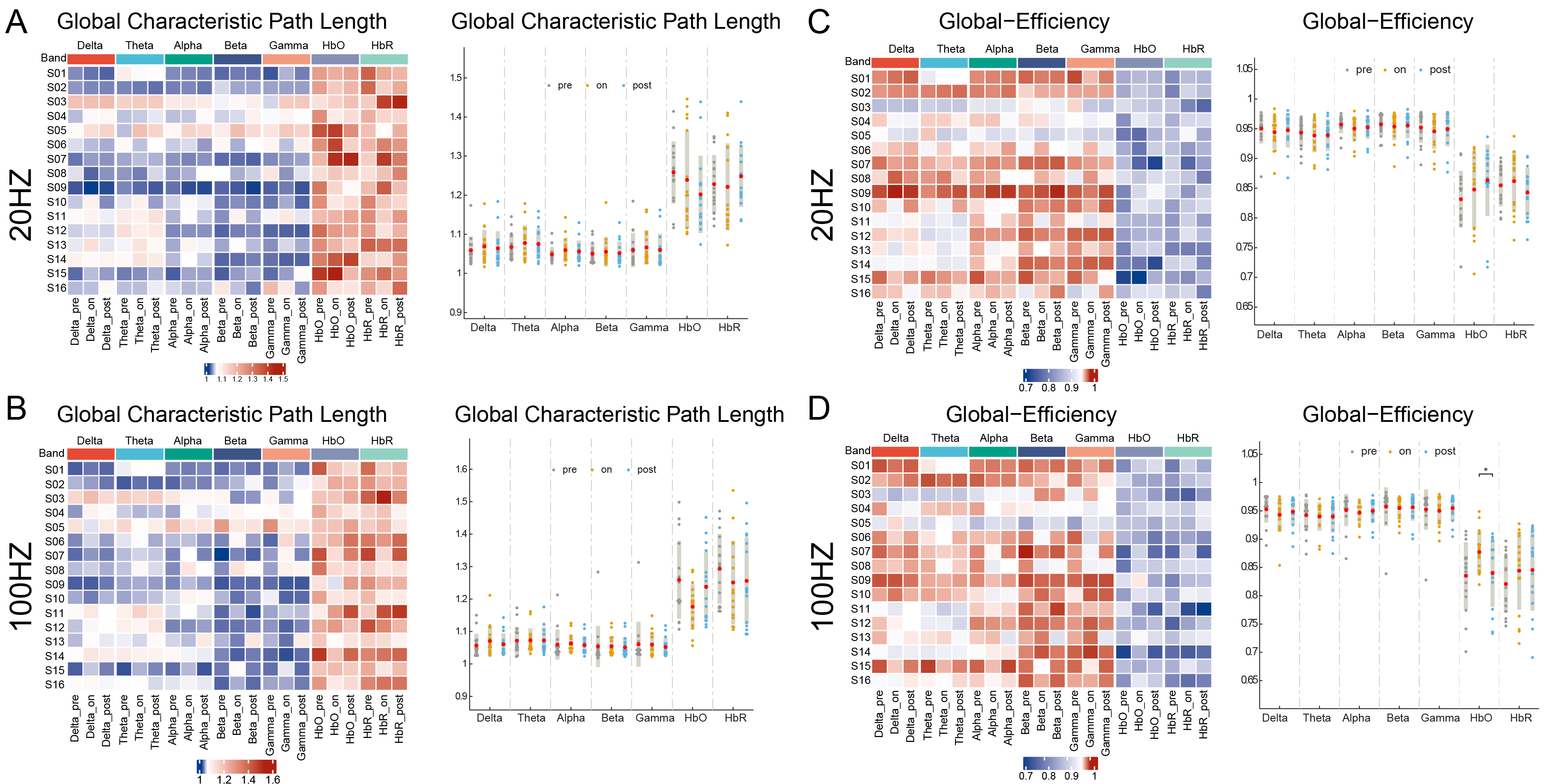

Supplement: Supplementary 1 — Figs. S1 to S3 Table S1 [file cbsystems.0539.f1.zip › S2.tif]

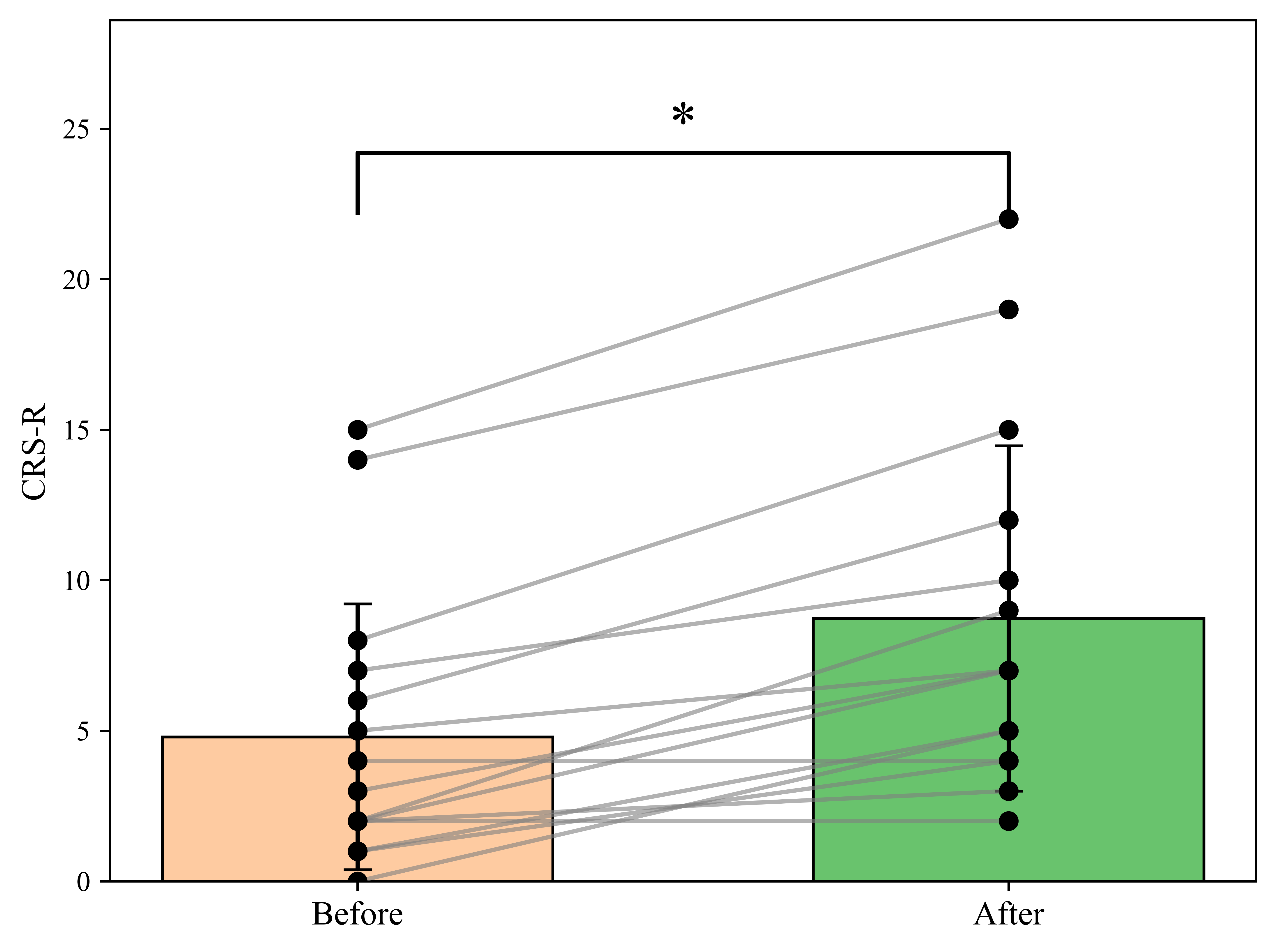

Supplement: Supplementary 1 — Figs. S1 to S3 Table S1 [file cbsystems.0539.f1.zip › S3.png]
